# Cell-particles interaction – selective uptake and transport of microdiamonds

**DOI:** 10.1038/s42003-024-05974-4

**Published:** 2024-03-13

**Authors:** Armin M. Ebrahimi, Wojciech Gawlik, Adam M. Wojciechowski, Zenon Rajfur

**Affiliations:** 1 Marian Smoluchowski Institute of Physics, Jagiellonian University, 30-348 Kraków, Poland; 2 Doctoral School of Exact and Natural Sciences, Jagiellonian University, 30-348 Kraków, Poland; 3 Jagiellonian Center of Biomedical Imaging, Jagiellonian University, 30-348 Kraków, Poland

**Keywords:** Endocytosis, Myosin

## Abstract

Diamond particles have recently emerged as novel agents in cellular studies because of their superb biocompatibility. Their unique characteristics, including small size and the presence of fluorescent color centers, stimulate many important applications. However, the mechanism of interaction between cells and diamond particles—uptake, transport, and final localization within cells—is not yet fully understood. Herein, we show a novel, to the best of our knowledge, cell behavior wherein cells actively target and uptake diamond particles rather than latex beads from their surroundings, followed by their active transport within cells. Furthermore, we demonstrate that myosin-X is involved in cell-particle interaction, while myosin-II does not participate in particle uptake and transport. These results can have important implications for drug delivery and improve sensing methods that use diamond particles.

## Abstract (web summary)

Fibroblasts actively search for and uptake microparticles from their environment and cells preferably uptake microdiamonds over latex beads. Myosin-X, EEA1 and microtubules are involved in this process.

## Introduction

Diamond particles are carbon-based materials^1^ that have recently been utilized especially in medical research as drug carriers^2,3^ and nano-sensors of physical quantities, including magnetic field and temperature in biological systems^4–6^.

Diamond nanoparticles are highly biocompatible^7,8^, which makes them useful for medical purposes and biological applications as antimicrobial agents, novel diagnostic approaches, or treatments of infectious diseases^9^. They can be utilized in gene/drug delivery^10^, nanoscale magnetometry^11^, label-free imaging^12^, and as live-cell nanothermometers in biosensing^13^. Owing to the chemical inertness and large surface area of diamond particles and the possibility of surface functionalization with medical and biological agents, nanodiamonds can play a remarkable role in drug delivery^14^ and microdiamonds (MDs) are also promising for medical applications^15,16^. However, the common issue with biological samples is that MDs need to be delivered inside cells. Thus, it is necessary to examine the possible effects of MDs on cell functioning, such as uptake, particle transport, and final localization.

Some diamonds have defects in their crystal lattice structure, called color centers, which are responsible for their strong and specific fluorescence. The most known type is the negatively charged nitrogen-vacancy (NV) center, which accounts for the highly stable red fluorescence of MDs (λ_em_ = 600–800 nm) when excited with green light (λ_exc_ = 510–565 nm)^17^. Importantly, the NV center has a ground-state spin (S = 1), which interacts with external magnetic fields. Hence, with the use of optically detected magnetic resonance imaging, the NV center can serve as a sensor to measure quantities such as magnetic field, temperature, and pH in biological systems, including cells^1,18^. Moreover, owing to its spin-related paramagnetism, this type of diamonds offers additional functionality useful for optically detected magnetic resonance and magnetic resonance imaging^19^.

When nano- or MD particles are present in an extracellular medium, two important cellular processes occur: particle uptake and subsequent internal relocation. The significance of uptake^20^ is underscored by the fact that it regulates the amount of particles inside cells. Similarly to various studies on nano-diamonds in cells^21–24^, research on the uptake of MDs^16,25,26^ can lead to the identification of novel approaches to improve the efficiency of drug treatments with diamond particles^27^. For this reason, our research focus on microdiamonds was driven by several factors. One key aspect is their size-dependent interaction, and because of their bigger size than of popular nanodiamonds, it is more convenient to detect their interactions within cells using optical microscopy. Our aim was to understand MDs’ biological impact, a crucial step in assessing their potential as cellular delivery platforms or sensors. Additionally, differences between MDs and nanodiamonds in cellular interaction were considered, including distinct physical properties and the study of macroscopic transport dynamics of MDs within cells. Furthermore, the larger size of MDs enhances imaging capabilities, facilitating real-time visualization in live cell experiments.

Cytoskeletal motor proteins, which convert chemical energy into mechanical forces, are known to play a crucial role in cellular mechanobiological processes, such as cargo transport (e.g., proteins) within cells^28,29^. This study focuses on the involvement of two motor proteins, myosin-X and myosin-II, in the uptake and internal transport of MDs and latex beads (LBs) to investigate their involvement in larger cargo transport within cells, in addition to their participation in protein transport^30–32^. Myosin-X^33–36^ is one of the unconventional myosins known to be bound to actin for movement and has binding sites for microtubules (MTs) in its tail^37^. This protein is important in the formation of filopodia and localizes at its tips^38–40^. Owing to its ability to generate mechanical force, myosin-II is connected to several cellular processes, such as filopodia formation, cell migration, and cell spreading^41–44^. However, the role of myosin-II and myosin-X in the uptake and transport of diamond particles is not yet fully understood. Furthermore, the relationship between myosin-X and MTs and actin in the presence of particles within cells is also not well known.

Here, we evaluated whether cells target particles in their environment and selectively uptake MDs over LBs. We also investigated the role of myosin-II and myosin-X in particle uptake and transport in cells and the relationship with cytoskeletal components. We show that myosin-II is not vital for particle uptake and transport in cells, while myosin-X shows higher accumulation around particles, pointing to its participation in cell-particles interaction. These results can have important implications in drug delivery by helping increase its efficiency and providing information essential in understanding cell functions, cell–virus interactions, and control of intracellular trafficking pathways.

## Results

### Active targeting, selective uptake, and active transport of MDs by cells

**Fig. 1:**
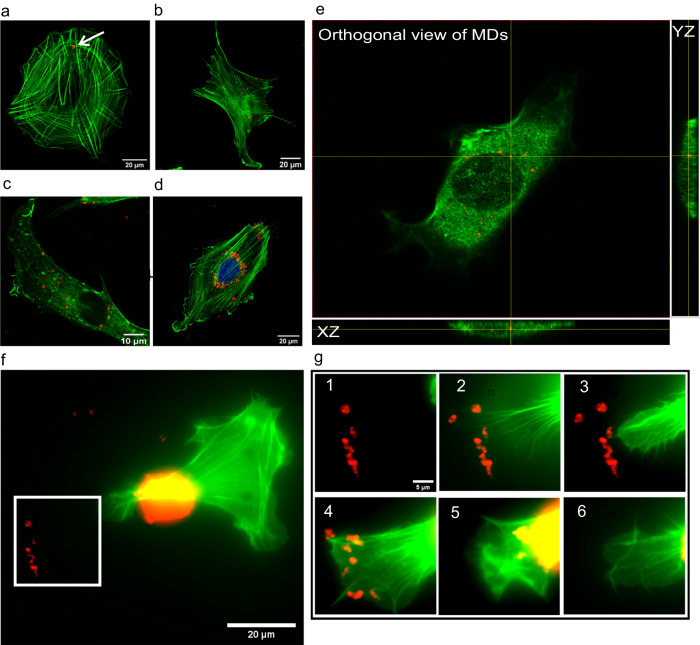
The amount of microdiamonds increases in cells with time. **a** Few microdiamonds (MDs) (red) are inside the cell (green) after 0.5 h of incubation. (**b**), **c** After 3 and 6 h, the amount of MDs increased with the incubation time. After 24 h (**d**), most MDs accumulate around the nucleus (blue). **e** Orthogonal side view of a cell with MDs inside the cell. Replications and number of cases for (**a–d**) are 6 and 45, respectively. **f** Live transfected cell with EGFP–F-tractin at the beginning of uptake. **g** Panels 1 to 6 are the time frames of the magnified area, marked as a white square in (**f**). Panels 1 and 2 show how the cell finds MDs employing protrusions: the cell extends the protrusions and creates lamellipodia to probe the particles (3,4). Finally, it is displayed in (5,6) that how the cell uptakes the particles inside. This process happens 16 h after adding the particles, and the time interval between images is 15 min. Replications and number of cases for (**f–g**) are 3 and 15, respectively.

Initially, the uptake process of MDs randomly sedimented on the surface of the culture plate by MEF cells was evaluated. Figure 1a–c show that the amount of particles collected by cells increased with incubation time, and Fig. 1d indicates that the amount of MDs around the cell nucleus was much larger after 24 h. The results for the same experiment with LBs can be seen in Supplementary Fig. 1. The orthogonal view mode of confocal imaging confirmed that the particles were inside the cells (Fig. 1e).

Subsequently, we performed 24-h live-cell imaging to visualize the interaction between cells transfected with EGFP–F-tractin and diamond particles, particularly the uptake process. Figure 1f and g show that the cells searched for particles employing their protrusions and then uptook MDs with their active extensions. The time evolution of this process is shown in Supplementary Video 1. The results of live-cell experiment with LBs can be seen in Supplementary Fig. 2.

**Fig. 2:**
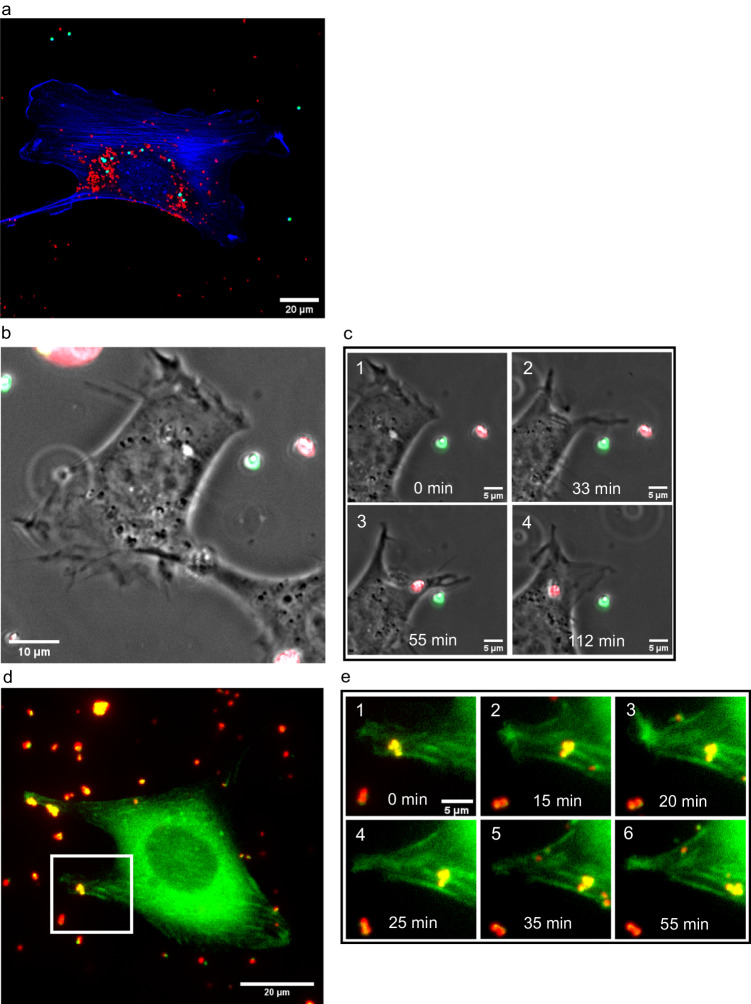
Cells selectively uptake microdiamonds than latex beads, and actively transport particles through actin filaments. **a** Cells with both microdiamonds (MDs) (red) and latex beads (LBs) (green) after 24 h of incubation, fixed and stained for nucleus and actin (both in blue), show more MDs than LBs inside the cells around the nucleus. Replications and number of cases are 4 and 70, respectively. **b** Full field of view of the cell at the beginning of uptake. **c** Time frames of the uptake of the MD by the cell which starts 1 h after adding particles to the sample show that the cell extends the protrusion to the MD to uptake it while leaving the LB outside the cell. In **c** (2), the cell is connected to the LB by protrusion, but it does not lead to the uptake of this particle. Replications and number of cases for (**b–c**) are 4 and 14, respectively. The results of selective uptake have been taken from a full field of view (300*300 µm),  one example can be seen in the Supplementary Fig. 4. **d** Full field of view of the investigated live cell transfected with EGFP-F-tractin and MDs deposited around it. **e** (1–6) The time frames of the cropped area marked in (**d**) show that the cells transport the MDs along the actin fiber; this process started 50 min after adding the particles. Replications and number of cases for (**d**–**e**) are 3 and 7, respectively.

There was a notable difference between the amount of MDs and LBs accumulated around the nucleus after 24 h of incubation, as shown in Fig. 2a. The concentration and surface functionalization were similar for both types of particles.

To study this, we employed live-cell imaging, with both MDs and LBs at the same concentration in the sample. The results of this experiment support the notion that even in cases of precipitation of both types of particles on the dish surface with a similar concentration (chance of uptake), cells show a higher tendency to uptake diamond particles to LBs. Figure 2b and c show the time frames of live-cell imaging of the preferable uptake of the MD while leaving the LB. The cell extends protrusion to the MD and LB but only uptakes the MD. The time course of this process is shown in Supplementary Video 2. A more detailed case of preferable uptake is shown in Supplementary Fig. 3, where there are more LBs next to a different cell; however, the cell uptakes only MDs.

In the next experiment, we studied the transport of MDs that have already been taken inside EGFP-F-tractin-transfected cells. Our results show that there is an active transport of MDs along actin filaments inside cells, as shown in Fig. 2d and e. The time course of this process is shown in Supplementary Videos 3 and 4.

In conclusion, our results suggest that cells search/probe for particles in their surroundings and preferably uptake MDs rather than LBs, and subsequently, cells actively transport MDs to the cell center. Active transport happens along actin filaments, suggesting that actin motor proteins can be part of the process of transport.

### Role of myosin-II in the search, uptake, and transport of particles

**Fig. 3:**
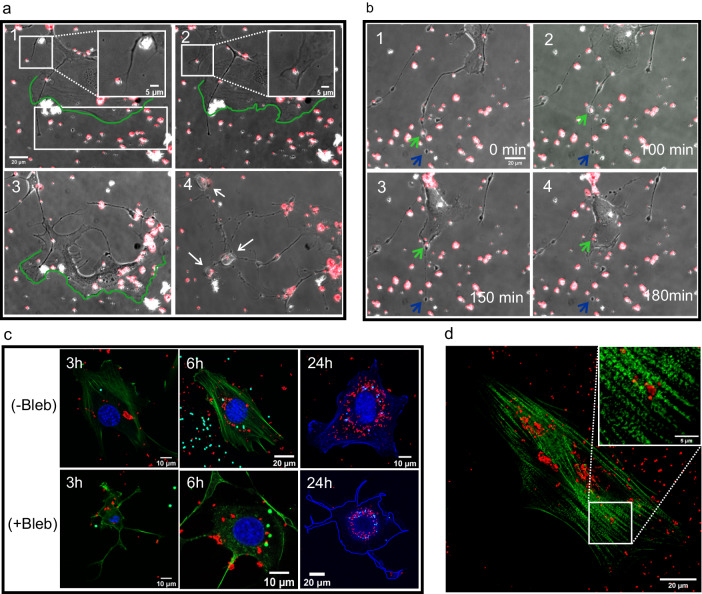
The processes of uptake and accumulation of particles around cell nucleus do not change when the activity of myosin-II is inhibited. (**a**) (1,2) Example of cells 2.5 and 3 h after Blebbistatin (Bleb) treatment. The cell uses the protrusions to find the microdiamonds (MDs) (red) on the dish surface. After 3 h, the cell extends the protrusion and forms lamellipodia to uptake MD inside. Further, there are MD particles attached to the dish surface as shown in the selected area (white rectangle) next to the cell membrane, outlined in green. After 8 h (3), the cell follows and reaches for MDs on the dish surface and finally probes that area and uptakes all of them by the active edge (green edge). In **a** (4), after 16 h, the same cell created many tails and vesicles, which are visible in the image. Interestingly, there are MDs present in those vesicles, as pointed to by white arrows. **b** Time frames of (1–4) show MDs located in the moving vesicles along the cell tail, entering the cell. The entire process takes 3 h and starts 2.5 h after adding Bleb. The movement of the vesicle together with the MDs is indicated by a green arrow. Finally, in **b** (4), the MD enters the cell. The tail has a fixed length throughout the process, and the end of the tail is shown by a blue arrow. Replications and number of cases for (a and b) are 3 and 27, respectively. **c** Fixed and stained cells with and without Bleb treatment after 3, 6, and 24 h of incubation. In both samples, the amount of MDs is larger than that of latex beads (LBs), but the amount of both particles does not change drastically in the Bleb-treated and normal cells. Finally, in the sample after 24 h, both MDs and LBs accumulate around the nucleus. (3, 6 h upper and lower panels: MDs in red, actin in green, LBs in cyan, nucleus in blue) and (24 h upper panel: MDs in red, actin and nucleus in blue, LBs in cyan –lower panel 24 h: MDs in red, LBs in green, artificially added cell edge in blue). Replications and number of cases are 3 and 55, respectively. **d** Cells incubated with MDs for 12 h were fixed and stained for myosin-II. The results indicate that myosin-II does not accumulate around particles, and it is distributed inside the cell similarly to control cells without particles and that myosin-II is distributed along actin fibers as we can see in Supplementary Fig. 6c-d. Replications and number of cases are 3 and 46, respectively.

Some proteins in the myosin family are known to be involved in the active transport within cells^29^. Herein, we investigated the role of myosin-II in cell probing, uptake, and transport of particles. We inhibited the activity of myosin-II with Blebbistatin (Bleb) to determine whether this treatment changes cell behavior. The experiment with fixed and live cells showed that although the cell morphology was different from untreated cells, there was neither notable change in the active search for particles by the cells (Fig. 3a) nor in the amount of MD particles taken inside the cells. The time course of this process is shown in Supplementary Video 5. The result of live-cell experiment with Bleb and LBs can be found in Supplementary Fig. 5.

Bleb treatment caused the cells to adopt a dendritic-shaped morphology and develop long extension. Furthermore, numerous vesicle-like structures are formed, which some of them can move along the tails and can contain single or multiple particles inside them. Note that the term vesicle-like in this paper was used specifically for the samples with myosin-II inhibition. Figure 3b shows that the MD is transported by those moving vesicles along the tails, which finally goes inside the cells. The time course of this process is shown in Supplementary Video 6. We also checked the effect of myosin-II inhibition on the amount of absorbed particles, their final location inside the cells, and their accumulation around the nucleus. Results (Fig. 3c) with fixed cells, both with and without Bleb, showed, cells still took up MDs and LBs similarly and accumulated them around the nucleus after 24 h.

Next, to study the role of myosin-II in particle transport inside cells, we fixed and stained cells for myosin-II. These experiments (Fig. 3d) showed that myosin-II distribution in the entire cell is similar to that in untreated cells and there was no noticeable change of its distribution in the presence of MDs. Also, in Supplementary Fig. 6b we can see that the distribution of myosin-II along actin fibers does not change when MDs are inside the untreated cells, compared to cells without particles. The results of control cell samples with Bleb are shown in Supplementary Fig. 7.

Taken together, there was almost no difference in particle uptake inside Bleb-treated cells and control cells, and most particles accumulated around the nucleus after 24 h of incubation. The amount of MDs was similar between the Bleb-treated cells and normal cells. The amount of LBs was also similar between the treated and normal cells. Moreover, we confirmed that cells can recover after washing the Bleb from the sample (Supplementary Fig. 8). Since we observed active particle transport in the cells, we expected that another motor protein from the myosin family may be involved in this process. Therefore, we investigated the possible involvement of myosin-X in the active transport of particles and its relationship with endosomes and cytoskeleton when particles are present in cells.

### Accumulation of myosin-X around particles within cells and its relationship with cytoskeleton

Since myosin-II did not seem to be directly related to the effects described above, we studied the possible role of myosin-X in cell–particle interaction, especially particle transport within cells. The experiment for untreated and Bleb-treated cells showed that myosin-X accumulated around some of the particles inside the cells. When the cells changed their morphology and created vesicles along their tails after Bleb treatment, myosin-X was present in those moving vesicles, and some of the vesicles contained particles inside them. Furthermore, myosin-X accumulated along the MTs in the area where the particles were located inside the cells.

**Fig. 4:**
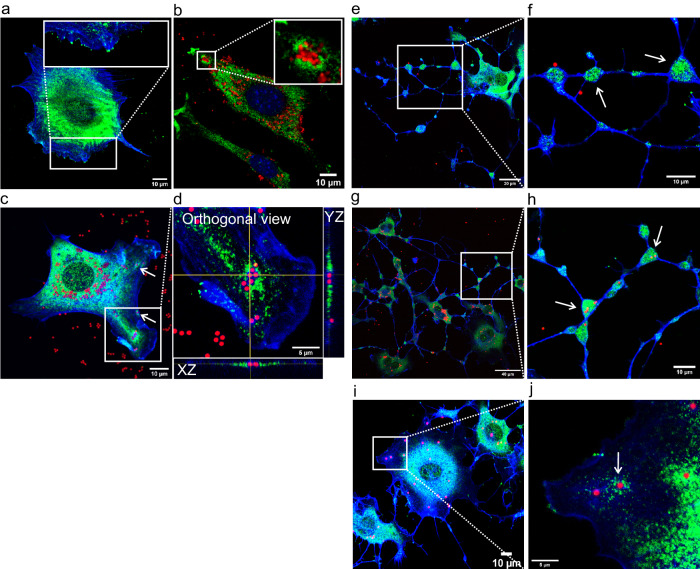
Myosin-X accumulates around the particles within normal and Bleb-treated cells. **a** Control cell without particles and without myosin-II inhibition, stained for myosin-X (green) and actin (blue) shows a normal distribution of myosin-X protein in the cell and at the tips of filopodia. **b** Results of untreated cells incubated with microdiamond particles (MDs) (red). There is accumulation of myosin-X around MDs inside the cell. Replications and number of cases for (**a**, **b**) are 7 and 34, respectively. **c** Myosin-X accumulates around latex beads (LBs) (red) inside the untreated cell. There is a higher accumulation of myosin-X around a cluster of six particles than around two or a single particle. **d** An orthogonal view of (**c**) shows the aggregation of myosin-X around the particles in both the xz and yz directions. Replications and number of cases for (**c, d**) are 3 and 7, respectively. (**e)** and (**g**) Bleb-treated cells show myosin-X in the vesicles (white arrows in **f** and **h**) along the cell tails after treatment for 6 h and 24 h, respectively. **i**, **j** Accumulation of myosin-X around the particles in cells after 6 h of treatment with Bleb. Replications and number of cases for (**e–j**) are 3 and 34, respectively.

The first observation described here (Fig. 4a) was that control cells, without particles and without myosin-II inhibition, stained for actin and myosin-X, showed that myosin-X localized at the end of the tips of the filopodia and in the center of the cell around the nucleus. In most cases, those cells did not show any specific accumulation of myosin-X in any part. Interestingly, untreated cells (without myosin-II inhibition), incubated with MDs or LBs (Fig. 4b-d) showed an increased accumulation of myosin-X protein around absorbed particles and a visible patch of myosin-X starting from the particles toward the cell center. Moreover, the amount of protein around the particles was related to the amount of the particles (as judged by the increased fluorescence of the labeled antibodies, (shown in Supplementary Fig. 9). Furthermore, we show that the accumulation of myosin-X around particles in cells does not depend on the type of particles and occurs for both MD and LB particles.

Next, we extended our myosin-X studies to the cells with myosin-II inhibition that were treated with Bleb (Fig. 4e-h). The goal was to check whether myosin-X can still accumulate around particles in cells with inhibited myosin-II activity and whether myosin-X accumulation occurs in moving vesicles (white arrows in f and h panels). We found that in Bleb-treated cells, myosin-X accumulated in all moving vesicles along the tails of the cell, and there were particles (MDs or LBs) inside most of those vesicles (confocal z-stack of vesicle with MD and myosin-X is shown in Supplementary Fig. 10). Since vesicles could confine MDs and move along the tails to the cells, the accumulation of myosin-X in them indicates that myosin-X could be involved in this process. Finally, as shown in Fig. 4i and j, when the cells were incubated for 6 h with Bleb and LBs, myosin-X densely accumulated around the particle in the selected area (white arrow in j panel). This result shows that the accumulation of myosin-X is independent of the activity of myosin-II.

**Fig. 5:**
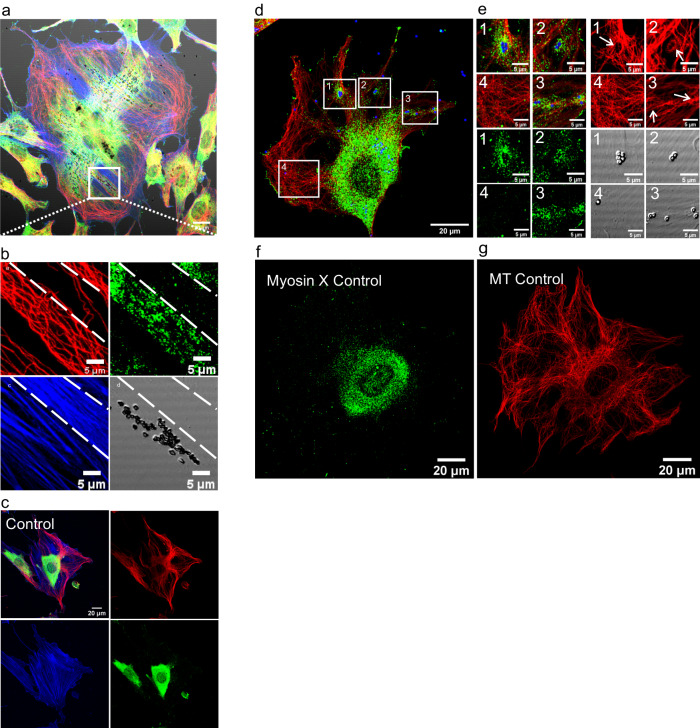
Myosin-X shows a colocalization with microtubules in the areas where particles are present. **a** Cross-staining for actin (blue), microtubules (MTs) (red), and myosin-X (green) in cells incubated for 24 h with non-fluorescent microdiamonds (MDs). Myosin-X is mostly localized with the MT in areas where there are MDs. Separated channels of this panel can be seen in Supplementary Fig. 12. **b** The cropped part of (**a**) shows that myosin-X is localized with MTs-rich areas, and not with actin reach areas (as shown by dashed lines). Replications and number of cases for (**a**, **b**) are 4 and 33, respectively. **c** The control sample without particles with the same staining shows no colocalization between the MT and myosin-X in the cell in the center of the image. **d** Evaluation of the accumulation of myosin-X (green) around latex beads (LBs) (blue) in four selected areas with microtubules (MT) (red) shows that in three areas, there is myosin-X around visible LBs inside the cell but shows no myosin-X in the last area without particles. **e** In the magnification of the four areas of image d for different imaging channels, the amount of myosin-X is smaller around one particle in area 3 than in area 1, which contains five particles. Interestingly, the amount of myosin-X is significantly reduced in area 4, even with the denser MT but without beads. Separated channels of panel d can be seen in Supplementary Fig. 13 where we can see myosin-X tracks along MT. Replications and number of cases for (**d**, **e**) are 3 and 10, respectively. **f** and **g** The control sample with the same staining as image d does not show any accumulation or colocalization of myosin-X and the MT without the presence of particles.

We investigated the distribution of myosin-X and the organization of cytoskeleton components such as actin and MTs when particles are present inside the untreated cells. Our data (Fig. 5) revealed that there was a higher myosin-X concentration specifically along the MT fibers in the areas where (non-fluorescent) MDs were absorbed. This phenomenon was observed for MDs with carboxylated or oxygenated (Supplementary Fig. 11) surface functionalization. These results suggest that there is a spatial and possibly functional relationship between myosin-X and MTs in cells that interact with particles. Figure 5a and b shows an example of this process for carboxylated non-fluorescent MDs and the cytoskeleton components such as actin and MTs (The separated channels of this panel (a) can be seen in Supplementary Fig. 12). Dashed lines separate the actin-rich and myosin-X-poor areas. The experiment showed that myosin-X was not specifically localized to the entire cytoskeleton but was localized to the MTs only in the areas with absorbed particles. A similar result with LBs is shown in Fig. 5d– (Separated channels of this panel can be seen in Supplementary Fig. 13). On the contrary, in the control sample without particles (Fig. 5c [one spread cell in the center], f, and g), cells exhibited diffused localization of myosin-X in all volumes, and there was no increased concentration of myosin-X along the MT. These results show that the accumulation of myosin-X along MTs depends on the presence of particles in those areas and indicates a possible relationship between myosin-X and the MTs when the particles are inside the cells.

### Investigation of the relationship between myosin-X and endosome protein EEA1

Material transport inside cells is known to involve endosomes^45^. Herein, we investigated the relationship between EEA1 (specific endosome marker) and myosin-X employing the colocalization technique of these two proteins.

**Fig. 6:**
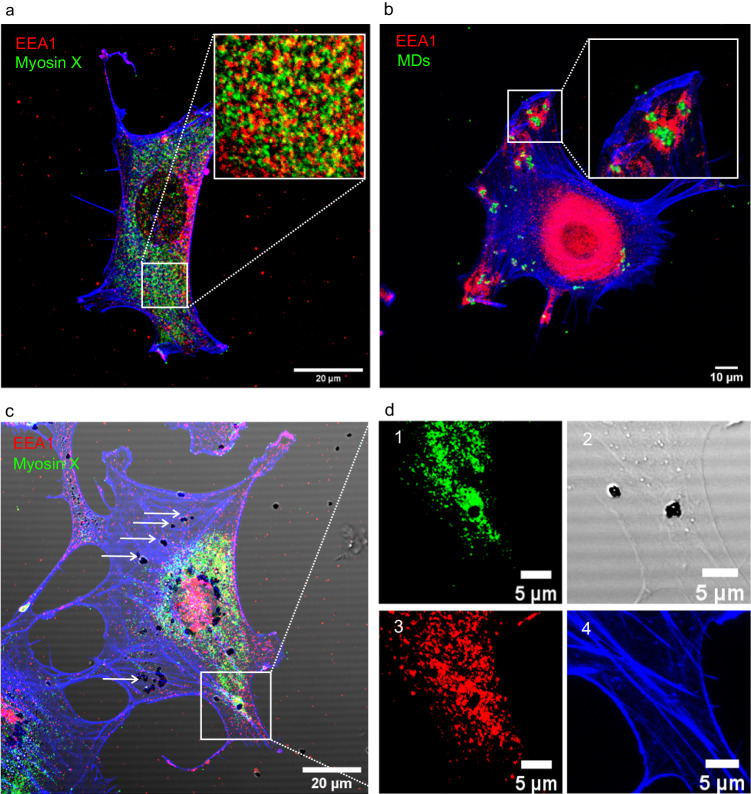
Myosin-X and EEA1 show colocalization around particles inside the cells. **a** Control staining of cells with EEA1 (red) and myosin-X (green) shows no accumulation or colocalization between these two proteins. **b** After 6 h of incubation of cells with microdiamonds (MDs) (green), the cells fixed and stained for EEA1 and actin (blue) show endosomes around MDs. **c** Cells after 12 h of incubation with non-fluorescent MDs fixed and stained for EEA1 (red) and myosin-X (green) show an accumulation of both proteins around MDs. The white arrows indicate other MDs inside the cell, in which there is no separate accumulation of any of the two proteins. This result suggests a functional relationship between myosin-X and EEA1. **d** The magnified selected area in **c** shows a dense accumulation of myosin-X and EEA1 around MDs. Replications and number of cases for (**b–d**) are 4 and 33, respectively.

We grew control cells without beads and then fixed and stained them for myosin-X and EEA1. The control experiment (Fig. 6a) showed no accumulation and no specific colocalization between both proteins. Thereafter, we checked whether endosomes formed around the absorbed MDs. Figure 6b shows that EEA1 accumulated around the MDs inside the cell. The cells were then incubated with (non-fluorescent) MDs and stained for myosin-X and EEA1. In several cases, the accumulation of both myosin-X and EEA1 around the particles inside the cell and their colocalization could be clearly seen (Fig. 6c). Interestingly, the accumulation of both EEA1 and myosin-X occurred only for some MDs in the cell (Fig. 6c and d), but when one of the proteins was not present around the particle, the other was also absent. These results indicate that there can be some functional relationship between these two proteins when particles are inside the cells.

## Discussion

The study of molecular mechanisms regulating the cell–micro/nanoparticle interaction is of particular importance in understanding many phenomena, such as endocytosis, viral entry into cells, or harmful effects of environmental pollutants. Because of the growing interest in nanoparticle and MD applications, we studied the initial stages of the cell uptake, and showed that cells tend to uptake more MDs in comparison to LBs. Also, we observed active transport of MDs along actin filaments and showed that some actin motor proteins can be a part of this internal transport mechanism.

We noticed that after 24 h of incubation of cells with MDs, most absorbed particles accumulated around but not inside the nucleus. Moreover, when cells were incubated with MDs and LBs in similar concentrations, there was a noticeable difference between the amount of MDs and LBs within the cells accumulated around the nucleus. This indicates that cells exhibit a higher uptake of MDs compared to LBs under the same conditions. There could be several factors resulting in higher uptake of MDs, one of which could be a different rate of particle sedimentation due to the different particle densities between MDs (3.52 g/cm^3^) and LBs (1.05 g/cm^3^). This can cause faster sedimentation of MDs, consequently resulting in a higher chance of finding MDs than LBs by cells. In the control experiments, we found slightly more sedimentation of MDs than of LBs on the cell dish surface (Supplementary Fig. 14). Even then, when the cells encountered MDs and LBs sedimented on the dish surface, the cells uptook mostly MDs and left LBs outside. Another factor can be related to the carbon-based nature of the diamond particles, which cells can detect as a more biocompatible material and uptake preferably. These results may have profound implications in drug delivery and cell–virus interaction.

A higher uptake of MDs could be a consequence of other factors, such as the stiffness, shape, and surface charge of particles^46^. Studies have shown that cells tend to uptake stiffer (MPa) particles more than softer (KPa) ones^47^. In our work, we used particles with the stiffness of diamonds (TPa) and polystyrene (GPa). It is unclear whether cells can detect this difference; therefore, further investigations are needed to determine whether this factor yields a higher MD uptake. Moreover, the uptake rate has been shown to change with different shapes of particles. For example, spherical gold particles are absorbed more than rod-shaped ones^48^, while this dependence is reversed with polystyrene^49^. Herein, we used round LBs and irregularly and randomly shaped MDs. Previous studies have shown that cells interact differently with round (35 nm–150 nm) or irregularly shaped nanodiamonds^50,51^. In terms of MDs, so far there are no previous data on the uptake of round microdiamonds. For now, it is unclear whether the shape (round or irregular) is causing a different uptake rate between MDs and LBs.

Next, because of the negative charge of cell membranes, particles with a positive surface charge have been shown to be absorbed more^52^. In the present study, both MDs and LBs were functionalized with carboxyl groups; hence, their surface charge could be considered negative. Moreover, to avoid MDs aggregation in cell samples in the live experiments for selective uptake, we pre-coated (MDs and LBs) with FBS^53^. To check the influence of FBS pre-coating on the observed phenomena (selective uptake), we performed the live-cell experiments with both FBS pre-coated MDs and non-FBS pre-coated MDs, similar to LBs particle, led to the same result.

Another factor in particle uptake can be related to the formation of corona proteins from cell media on the surface of particles^54^, which can lead to different corona composition on particles, that can affect their uptake.

Another finding is that cells actively search for both MDs and LBs on the dish surface and detect their presence using protrusions and filopodia and then target MDs for absorption and uptake them via lamellipodia. Our findings show that cells form protrusions in a similar manner when interacting with both MDs and LBs. This suggests that cells sense both types of particles, but in most cases, this sensing leads to the uptake of MDs, while LBs are not taken up. While our study did not specifically investigate whether the presence of MDs induces unique types of cellular protrusions, a more in-depth characterization of cellular protrusions in response to each particle type would require further research.

Moreover, by observing an active transport of MDs along actin filaments, we suggest that actin motor proteins can be part of the process of uptake and particle transport within cells.

One of the hypotheses was that myosin-II may be involved in this dynamic process. However, inhibition of myosin-II activity showed that the cells maintained their active targeting for MDs and LBs and uptook particles by active leading edges similar to the untreated cells. There was almost no difference between the amount of particles inside the cells and their accumulation around the nucleus for both Bleb-treated and untreated cells. Thus, the activity of myosin-II does not appear to be crucial for cells to search and uptake particles. Our results also confirm the biocompatibility of MDs in a concentration of up to 100 µg/mL as they neither cause apoptosis nor alter the morphology of MEF cells (Supplementary Fig. 15).

We then evaluated another myosin family member: myosin-X. Immunostaining of the cells containing absorbed particles showed an accumulation of myosin-X around those particles. To check whether there was any spatial relationship between the different components of the cytoskeleton and myosin-X in the presence of particles inside the cells, we stained the cells containing MDs for actin, MTs, and myosin-X. There was a clear myosin-X path from the center of the cell to the general area around the particle, but this was not observed in the areas where there were no beads and in the control cells without beads. The accumulation of myosin-X in areas with particles occurred where the MT structures are present. The control sample without beads showed that there was no accumulation of this protein with the MTs within most cells. We noticed that the amount of myosin-X around the particles increased with the amount of the particles.

However, these findings suggest that more experiments are needed to investigate whether MD’s transport is occurring via actin-based motors or MT-based motors. Similarly, one can investigate whether myosin-X acts as a transporter of the MDs or play other roles in MDs accumulation in cells with and without myosin-II inhibition.

Since it is known that endosomes, are involved in the transport pathway within cells^55^, we studied the possible interaction of myosin-X and endosomes. Our experiments showed colocalization between myosin-X and the EEA1 endosome protein around particles in the cells, which we did not see in control cells without particles. Moreover, whenever the particles were confined in the endosome, myosin-X also accumulated around them. These results suggest a possible functional relationship of myosin-X with endosomes and MTs, when particles are inside the cells.

Further investigations are crucial to enhance our understanding of myosin-X involvement in cellular processes and interactions with particles, including both particle uptake and intracellular active transport. Additionally, these studies will shed light on the interplay between myosin-X, EEA1, and MTs, detailing their accumulation dynamics around particles, and providing insights into the temporal evolution of these complex interactions.

## Methods

### Cell culture

The MEF 3T3 cell line (Mouse Embryonic Fibroblasts, ATCC CRL-1658) was grown in DMEM low glucose medium (Biowest, L0066), supplemented with 10% Gibco FBS (Thermo Fisher Scientific, 10270106) and 1% penicillin (Biowest, L0022), and maintained at 37 °C, 5% CO_2_, and 100% humidity. For cell passaging, after removing old DMEM, sample was washed with phosphate-buffered saline (PBS) (Sigma, D8537) and we added trypsin (Biowest, L0930) to cells and incubated them for 7 minutes at 37 °C. After that, cells were washed with 1 ml fresh DMEM to detach cells from surface, then 15% of cells were added to a new 25 cm² cell culture flask (Sarstedt, 83.3910) with 5 ml fresh media and kept in incubator.

### Diamonds and latex particles

One micron-sized carboxylated red fluorescent diamonds (NDNV1umCOOH, Adamas Nanotechnologies, Raleigh, NC USA) with approximately 3.5 ppm NV were used in this study. They were provided in an aqueous solution (1 mg/mL in DI water). Fluorescence was excited using the 561 nm laser line, and emission was detected at 620–700 nm using a confocal microscope.

Oxygenated 1 µm NV diamonds and carboxylated 1 µm non-NV diamonds were prepared by the Gdansk University of Technology group. Carboxylate-modified polystyrene LBs with 1 µm diameter (Sigma-Aldrich, L4655) were excited at 488 nm, and emission was detected at 500–550 nm. Carboxylate-modified polystyrene LBs with 1 µm diameter (Invitrogen, 2291691) were excited at 561 nm, and emission was detected at 600–650 nm. More characteristics of MDs, such as average size and zeta potential, as well as more information about LBs, can be found in the supplementary Note file in Characterization of particles.

To avoid aggregation of the diamonds in the cell media, first we pre-coated the MDs and LBs with FBS before the experiment. For pre-coating particles with FBS, MDs and LBs were added to separate 1.5 ml sterile Eppendorf tubes and left for sonication for 5 minutes. Then 10% pure FBS was added to the particles and left for 15 minutes at room temperature. After that, particles were added to the cell samples for the experiments.

It is worth to mention that the utilization of carboxylated (COOH) and oxygenated MDs with and without NV centers, and carboxylated latex beads in our study served different purposes. For instance, non-fluorescent MDs (without NV centers) were employed due to the limited number of available color channels on the confocal microscope. More information can be found in the supplementary Note file in Characterization of particles.

### Myosin-II inhibitor

To inhibit the activity of myosin-II, we used (-)-Blebbistatin (Sigma-Aldrich, B0560), a selective inhibitor of non-muscle myosin-II, in 1 mg powder dissolved in 0.5 mL dimethyl sulfoxide (DMSO) (Sigma, D8418) to reach the concentration of 2 mg/mL.

### Antibodies

Both primary and secondary antibodies were used with the concentration of (2 µg/ml in 3% BSA).

The following primary antibodies were used: alpha-tubulin (Sigma, T9026), myosin-II (Sigma, M8064), myosin-X (Sigma, HPA024223), EEA1 (Cell signaling, C45B10 and Sigma, E7659), LC3B (Invitrogen, PA5-32254).

The following secondary antibodies (R: anti-rabbit, M=Anti-mouse) purchased from Invitrogen and were utilized: Alexa Fluor 647 labeled (R: A21244, M: A32728), Alexa Fluor 488 labeled (R: A32731, M: A32723), and pacific blue goat (R: P10994). For actin staining, the cells were fixed, permeabilized, and incubated with Alexa Fluor 405 nm phalloidin (Invitrogen, A3J104) or atto 488 nm phalloidin (Sigma, 49409). For nucleus staining, we used Hoechst333422 (1:500 in PBS buffer) (Invitrogen H3570).

Triton X-100, Tween20 and formaldehyde were obtained from Sigma (T8787, P1379 and P6148), glycine from Merck (S5780213925) and bovine serum albumin (BSA) from Millipore (840851).

Plasmid: ITPKA-mNeonGreen^56^ was a gift from Dorus Gadella (Addgene plasmid #98883; http://n2t.net/addgene:98883; RRID: Addgene_98883).

### Cell labeling and immunofluorescence

The cells were fixed with 4% formaldehyde in PBS for 20 min at 37 °C and permeabilized for 5 min in 0.1% Triton X-100 in PBS at room temperature (RT 21 °C). Next, they were incubated with 0.1 M glycine in PBS for 10 min at RT. The samples were then washed three times with PBS at RT. For primary antibody staining, nonspecific sites were blocked with 3% BSA PBS buffer for 60 min at RT. The primary antibodies were applied to the samples overnight at 4 °C, and the samples were then washed three times with PBS for 10 min. Tween-20 was added (final concentration of 0.02%) to enhance effective washings, leading to decreased nonspecific background staining. The secondary antibodies were applied for 1 h at RT. For actin staining, the fixed cells were incubated with 405 or 488 nm conjugated phalloidin for 60 min. For nucleus staining, we used Hoechest3322 for 30 min. Finally, the samples were washed three times with PBS for 10 min and then imaged via confocal microscopy.

### Sample preparation

For the confocal and live-cell imaging experiments, the cells were seeded at 5000 cells per dish density and kept overnight in an incubator at 37 degrees in a 2 ml fresh medium and plated in 35 mm glass bottom dishes with 20 mm well, and a thickness of #1.5 (0.16–0.19 mm), designed for high-resolution imaging (Cellvis, D35-20-1.5-N). Thereafter, we added either fluorescent or non-fluorescent MDs and/or LBs and incubated the cells from 0.5 h up to 24 h depending on the experiment.

For confocal microscopy experiments, the cells were fixed and depending on the experiment, they were stained for actin and nucleus and indirect immunostaining for MTs, myosin-X, myosin-II, and endosomes. Finally, the samples were evaluated via confocal microscopy. For Blebbistatin experiments, after seeding and overnight incubation, we added Bleb at a final concentration of 20 µM in DMEM; then after 45 min, we added the particles. For live-cell imaging, during the experiment, the cells were kept in an environmental chamber as an incubator on the microscope stage to maintain the cells at 37 °C, 5% CO_2_, and 100% humidity. Live-cell imaging with EGFP–F-tractin-transfected cells was conducted for either 45 h (Fig. 1f) or 3h (Fig. 2d) and images were taken every 15 or 5 min via fluorescence microscopy, respectively. Time interval for live imaging (Fig. 2b) was 1.5 min and for Fig. 3 (a, b) was 10 min. The concentration of the particles (MDs and/or LBs) was 5 µg/mL for the experiments in Fig. 1f and g, Fig. 2b-e, Fig. 3a, b, and Fig. 6 and 10 µg/mL for the experiments in Fig. 1a–e, Fig. 2a, Fig. 3c, d, Fig. 4, and Fig. 5.

### Confocal and fluorescence microscopy and data analysis

Confocal images of the stained cells were acquired using Zeiss LSM 710 confocal module set on Zeiss Axio Observer Z1 (Carl Zeiss, Germany) using an oil immersion 40×1.4 NA plan-apochromat objective. In confocal imaging, three laser lines – 405, 488 and 561 nm - were used for excitation.

Fluorescence images of the live cells were taken using Zeiss Axio Observer Z1 (Carl Zeiss) with HXP 120 V and arc lamp (HXP R 120 W/45 C VIS, OSRAM), using an oil immersion 40×1.4 NA plan-apochromat objective and Hamamatsu ORCA-Flash 4.0 camera. For moving the stage and maintaining the focus, WSB PiezoDrive CAN and fc12 were used (Carl Zeiss), respectively. Heating Insert P Lab-Tek™ S (Pecon, Germany) together with TempModule S (Pecon, Germany) and CO2 Module S (Pecon, Germany) provided the right conditions for live cell imaging: 37 °C and 5% CO2. Green channel for latex beads and EGFP–F-tractin-transfected cells was observed using the standard Zeiss filter cube 38 HE (Ex: BP 470/40 (HE), Em: BP 525/50 (HE)). Fluorescence images of NV– diamonds were collected using a custom fluorescence cube built with: Band Pass Filter 470/40 nm (excitation), DMLP 567 nm dichroic mirror, and Long Pass 600 nm filter (emission).

For image analysis, Fiji (https://imagej.net/software/fiji/#publication) was used.

In order to decrease the background signal, for all of the images used in this paper we adjusted the contrast and channel’s intensity to have a clear presentation of the results. More information and raw image example can be seen in Supplementary Fig. 16. Moreover, to have a consistent color coding between the panels in the Figures, we changed the color of some components in the panels.

### Statistics and reproducibility

All experiments in this work were repeated at least in three independent experiments and the data is represented as cases that have been chosen among all the cases in each experiment to show the specific phenomenon. Detailed methods are provided to ensure the reproducibility of experiments. Cell numbers can be found in sample preparation section. Replicates (number of dishes) are noted in each figure legend as is the number of cases in which the studied phenomenon has been observed. For all the control experiments, replications and number of cases are 3 and 30, respectively.

### Reporting summary

Further information on research design is available in the Nature Portfolio Reporting Summary linked to this article.

### Supplementary information

Supplementary Information

Description of Additional Supplementary Files

Supplementary Video 1

Supplementary Video 2

Supplementary Video 3

Supplementary Video 4

Supplementary Video 5

Supplementary video 6

Supplementary Video 7

Supplementary video 8

Supplementary video 9

Supplementary video 10

Supplementary video 11

Supplementary video 12

Reporting Summary

## Data Availability

Collected data supporting the findings of this manuscript are available from the corresponding authors upon reasonable request because of the large size of the microscopy image data. Raw image data were acquired and stored in CZI format, developed by Zeiss. Additional data are provided in Supplementary Information.
