# Supplementary material for: Cell-particles interaction – selective uptake and transport of microdiamonds
Source: Commun Biol. 2024 Mar 13;7:318. doi: 10.1038/s42003-024-05974-4 (PMC10937934; doi:10.1038/s42003-024-05974-4)
Supplement: Supplementary file 1 — Supplementary Information [file 42003_2024_5974_MOESM1_ESM.pdf]

Supplementary Material

Interaction of LBs with cells

Latex beads with a diameter of 1 µm served as a positive control to study the interaction between microdiamonds (MDs) and cells. Supplementary Fig. 1a shows that the fixed and stained cells incubated with latex beads (LBs) contained LBs inside the cells (after 6 h). Fig. 1b shows a lower amount of LBs around the nucleus (after 24 h), compared to MDs. This could be a consequence of the less frequent uptake of LBs in the surrounding area as confirmed by live-cell imaging.

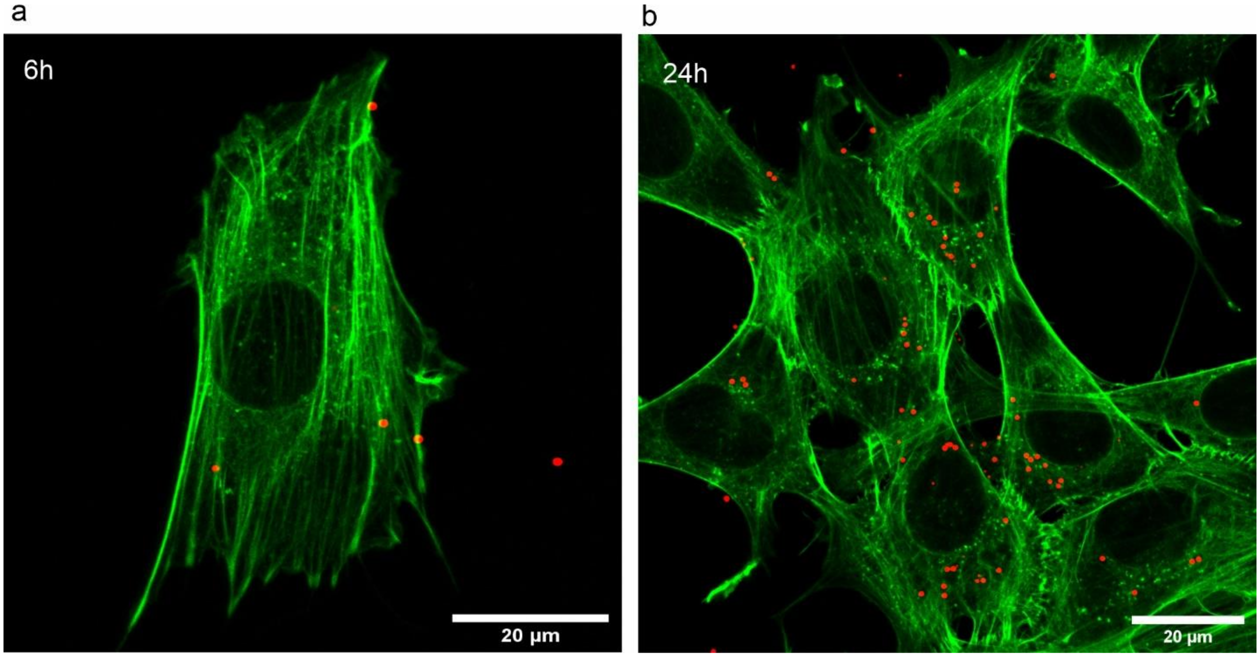

Supplementary Fig 1. Cells uptake latex beads in a lower amount compared to microdiamonds. The cells incubated with latex beads (LBs) after (a) 6 and (b) 24 h of incubation show a smaller amount of particles within the cells themselves. The accumulation of LBs around the nucleus is lesser than that of microdiamonds after 24 h. Replications and number of cases are 3 and 30, respectively.

Supplementary Fig. 2 shows one cell during 4 h of live-cell imaging with only LBs in the media. Even when the latex beads were close to the cells, the cells were much less active in terms of uptake of the LBs than of the MDs (Fig. 1f). The time course of this process is shown in Supplementary Video 7.

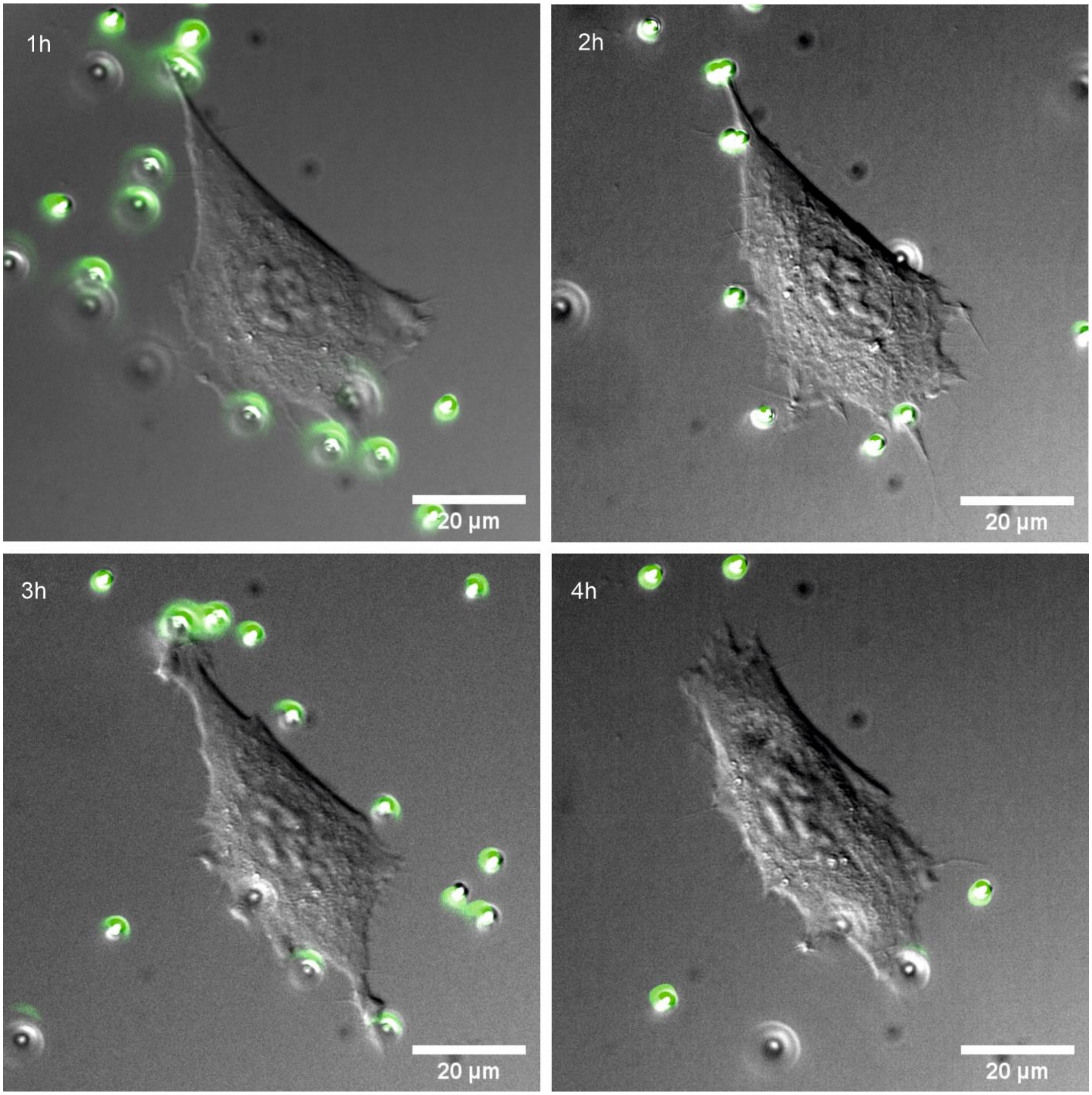

Supplementary Fig. 2. Live-cell imaging of cells with latex beads shows a low interaction between them. The cell during 4 h of live-cell imaging shows a low interaction with latex beads. Replications and number of cases are 3 and 20, respectively.

Additional example of the preferable uptake of MDs over LBs

Supplementary Fig. 3 (left panels a to d) shows an additional example of the preferable uptake similar to that in Fig. 2b. In the left panel, there are four frames, with each having four marked selected areas: Green LBs are in the areas marked 1, 2, and 3 and MDs in the area marked 4. Panel a shows the beginning of the uptake in the four selected areas. There are three LBs in area 1 set on the surface, and the cell is touching them with its protrusions. Area 2 shows another LB next to the cell edge, and area 3 shows another LB next to the cell protrusions that interact with it. Finally, in area 4, there is an MD cluster next to the cell, but there are no visible protrusions leading to it. Panel b shows the cell after 20 min: The cell moves closer to area 4 (MD) and extends protrusions to the MDs. Simultaneously, the other three areas with LBs remain the same, and the cell does not tend to interact actively with them. Therefore, neither the cell edge nor the protrusions play a role in the absorption of LBs. In panel c, the MD cluster is almost engulfed by the cell membrane, whereas the LBs in the other three areas with protrusions in the vicinity are still at the same place. Finally, in panel d (after 60 min), the MDs are already inside the cell, while the LBs are still outside the cell in the other three areas. The cell is in contact with the LBs with its protrusions throughout the process. The right panels show the magnified images of the four selected areas from a to d for a better understanding of the time course of this process, which happened within 1 h from a to d. This result shows that although there are five LBs on the surface next to cells, the cell prefers to uptake diamonds (at a ratio of 1 [MD]: 5 [LBs]) and not expand the interaction with LBs to absorb them. The time course of this process is shown in Supplementary Video 10.

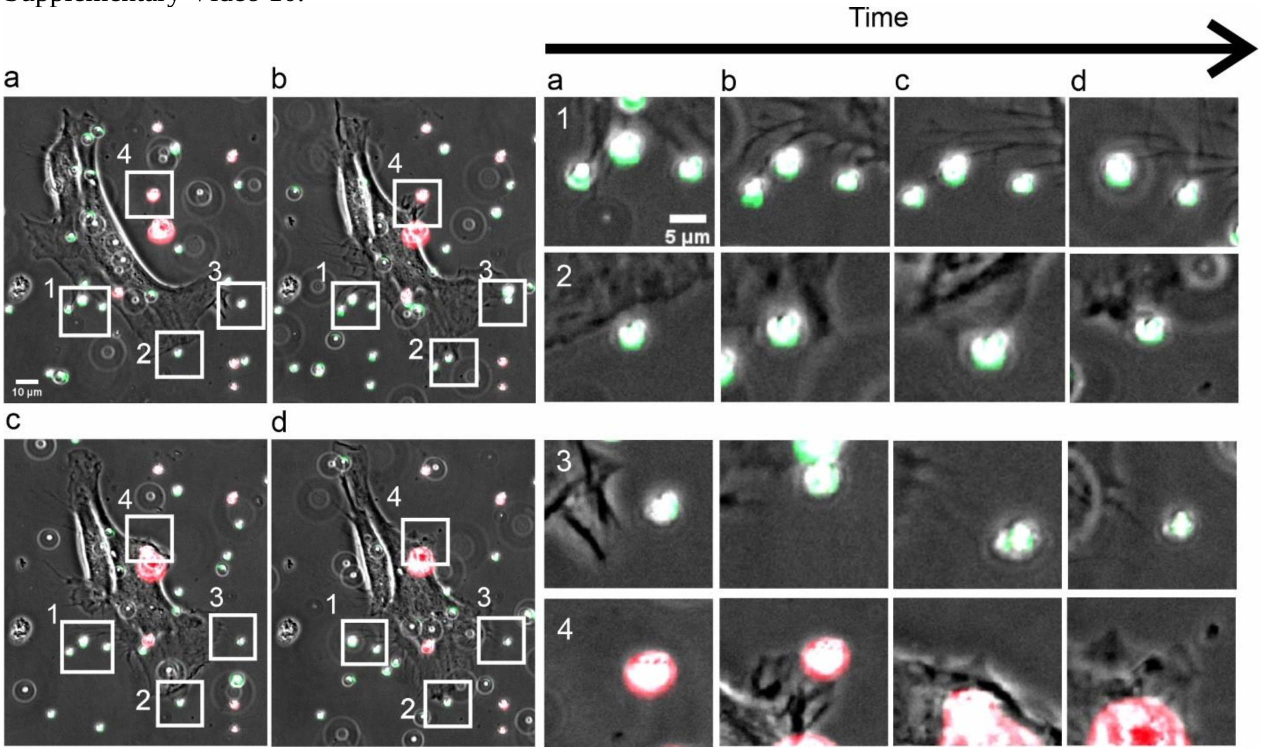

Supplementary Fig. 3. Additional example of selective uptake of microdiamonds over latex beads by cells. (a-d) Preferable uptake of microdiamonds (MDs) by cells. The left panels from a to d show the time frames of this process. In each panel, there are four marked areas. In areas 1, 2, and 3, there are latex beads (LBs) next to the cell, and in area 4, there are MDs. Over time, the cell does not absorb LBs and, while approaching diamond particles and extending protrusions, uptakes MDs inside.

The results of our live-cell imaging experiments have been taken from different time lapse sequences with a field of view size of ~300 µm x 300 µm. Supplementary Fig. 4. Shows a representative single image from a full field of view of a live-cell time lapse sequence.

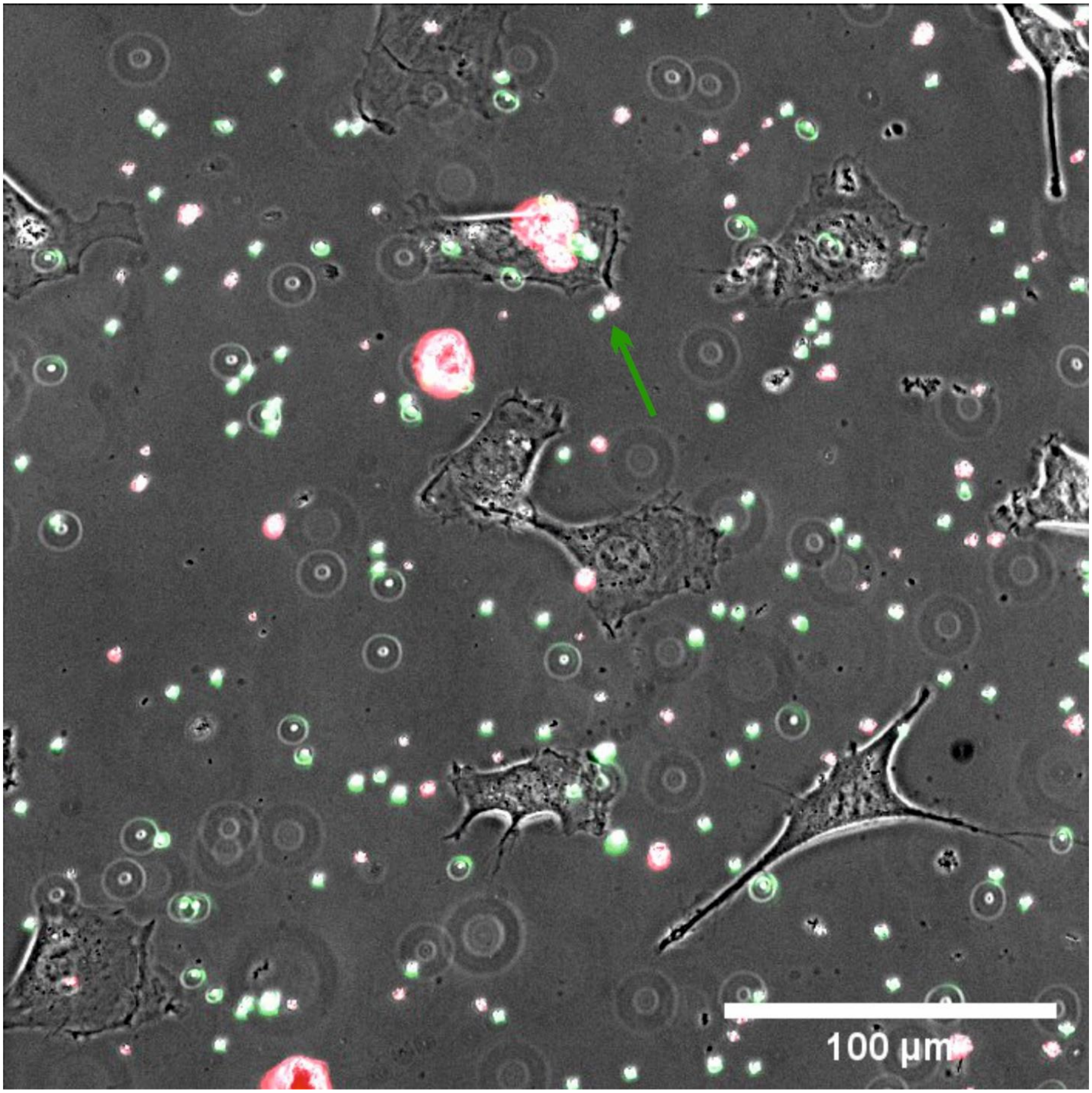

Supplementary Fig. 4. Full field of view of the live-cell experiments (~300*300 µm). The green arrow points to the cell where we observed selective uptake of MDs (red).

Blebbistatin treatment has no influence on LB uptake

To eliminate the possible influence of MDs on the uptake of LBs, we performed the experiments only for the cells incubated with LBs. We observed that with 20 μM of Blebbistatin (Bleb), the cell uptake of LBs were lower than those of MDs, similar to normal cells without Bleb. The results are presented in Supplementary Fig. 5, which shows two different cells (a and b after 2, 4, and 8 h) with LBs around them. Therein, the cells changed their morphology owing to Bleb treatment and formed long extensions, but even after 8 h, there was no improvement in uptaking particles. The time course of this process is shown in Videos Supplementary Video 8 and Supplementary Video 9.

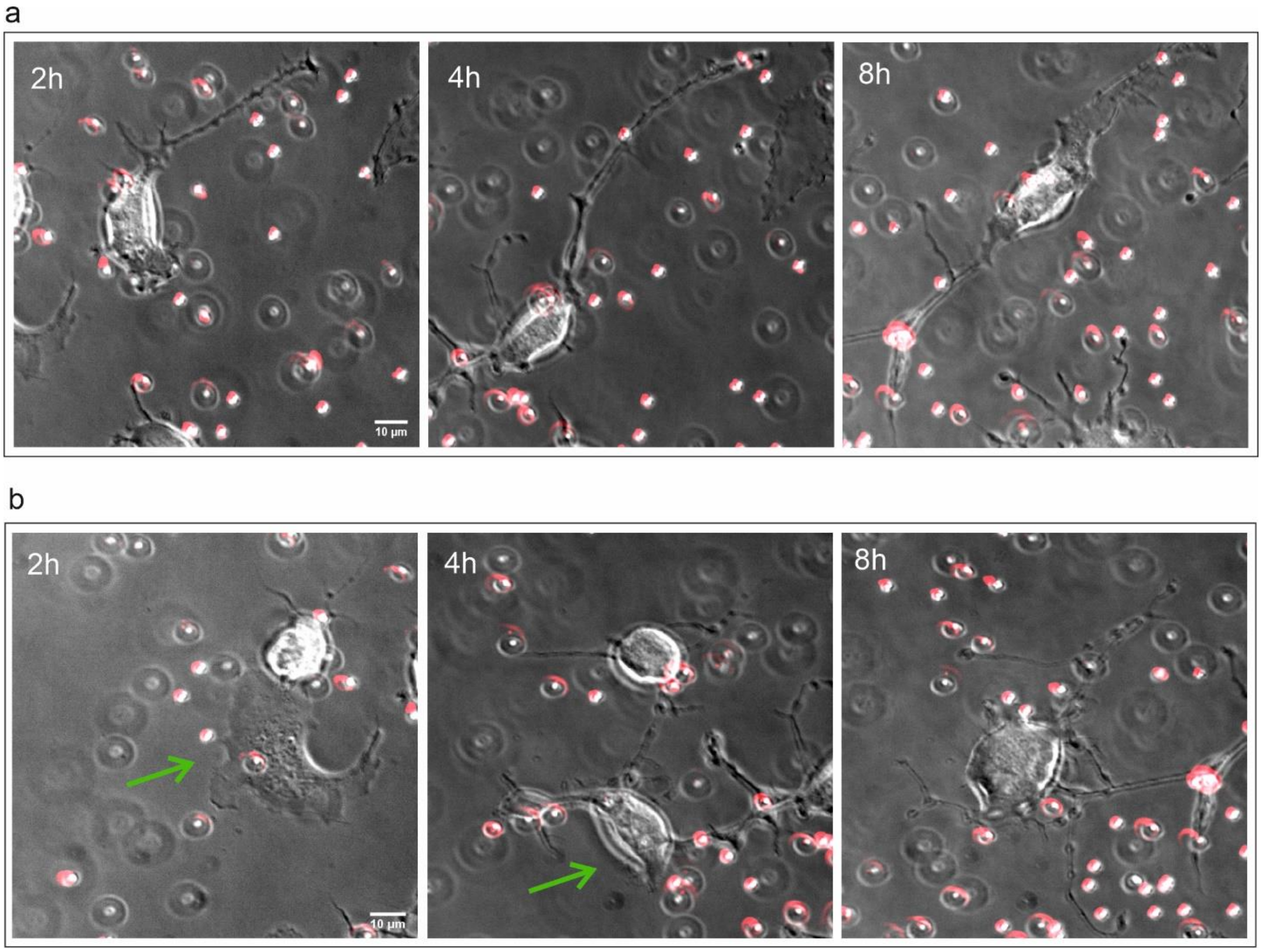

Supplementary Fig. 5. Live-cell imaging of Bleb-treated cells with latex beads shows low interaction between them. Blebbistatin-treated cells do not show interaction and uptake as high as those with microdiamonds. (a) A cell after 2, 4, and 8 h with latex beads. (b) Another cell after 2, 4, and 8 h. The green arrow points to the cell of the study. Replications and number of cases are 2 and 30, respectively.

Control cell sample with myosin-II

An example of a control cell without particles stained for myosin-II is shown in Supplementary Fig. 6a. The experiment showed that there was no difference between myosin-II distribution with and without particles in the cells.

Also, Supplementary Fig. 6b exhibited a distribution of this protein in cells grown with MDs and from c and d we can see that it colocalized along actin filaments, which is in agreement with the literature.

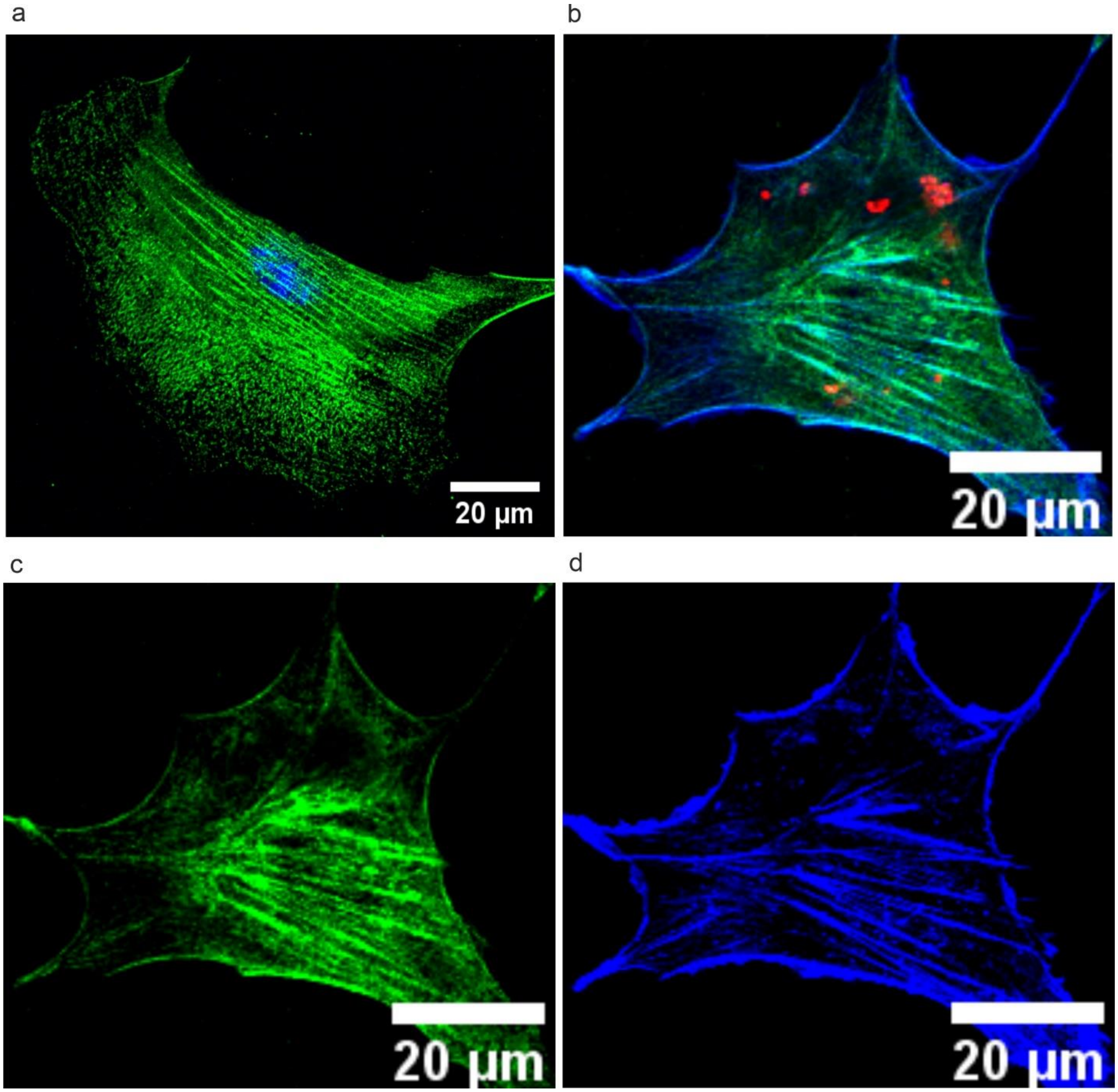

Supplementary Fig. 6. Fixed cells stained for myosin-II show normal distribution of myosin-II in cells with particles, similar to control samples. (a) A control sample of myosin-II staining shows a normal distribution of this protein. (b, c, d) Show myosin-II distributed along actin filaments.

Control samples from cells with Bleb

Supplementary Fig. 7 shows three control cells: (a) 20 µM Bleb, (b) 100 µM Bleb, and (c) 20 µM DMSO (Bleb solvent). Supplementary Fig. 7a and b reveal the same morphology as in Fig. 4e and g. Further, 20 µM DMSO did not show any negative effect on cell viability after 6 h of incubation with DMSO. All samples were stained for MT (red), actin (blue), and myosin-X (green).

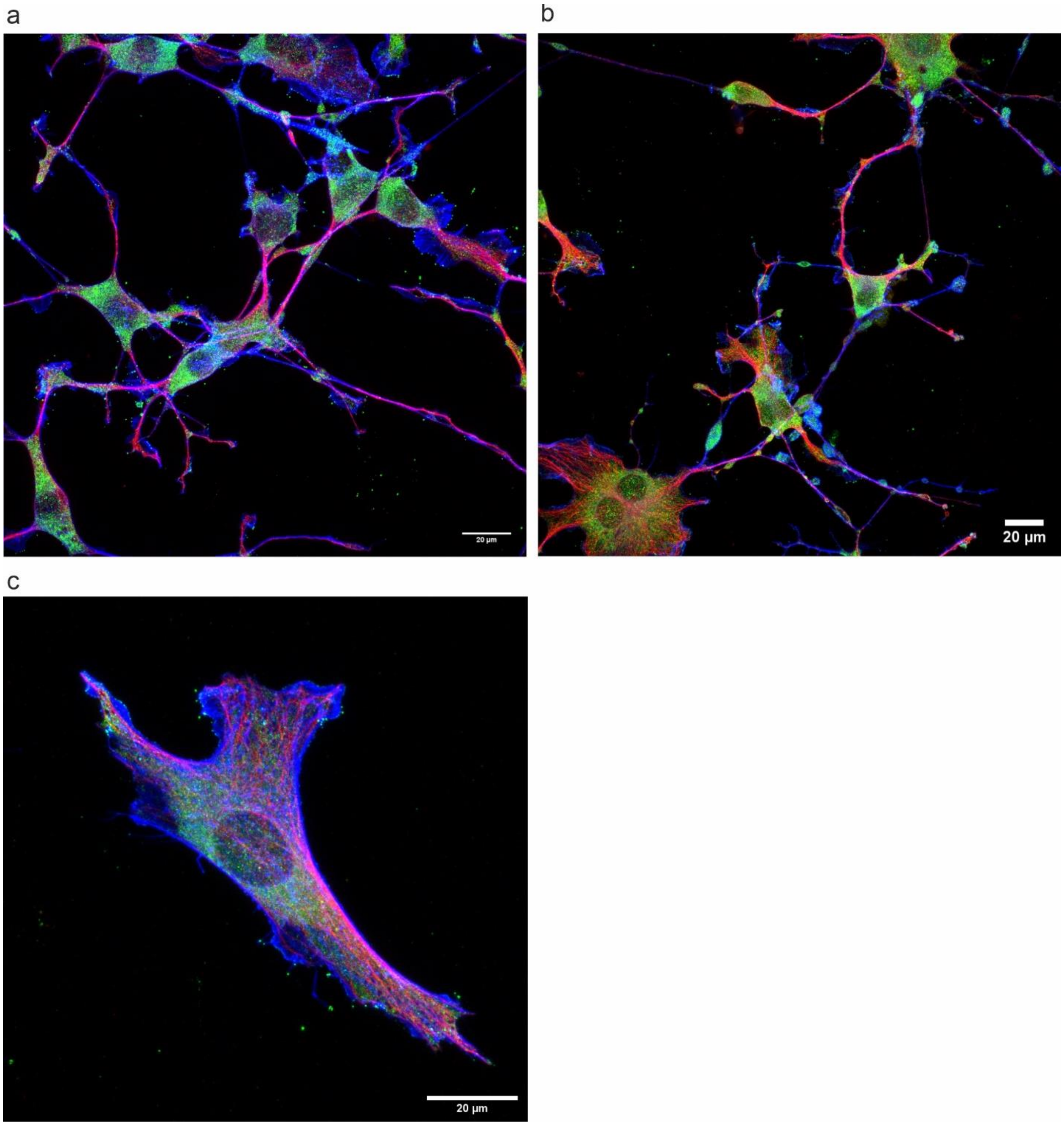

Supplementary Fig. 7. Control samples with Bleb and DMSO. (a), (b) Control samples with Blebbistatin (Bleb) (20 and 100 µM) show the cell morphology (actin in blue and microtubules in red) after Bleb treatment and myosin-X (green) distribution. (c) Cell sample with DMSO (20 µM) shows that this concentration of DMSO does not cause cell death or visible change.

In Supplementary Fig. 8 we can see a time-lapse of cells incubated with Blebbistatin for 6 h and then it was washed out. One can see that the cell can recover its morphology and start to retract the tails after recovering. The time course of this process is shown in Supplementary Video 12.

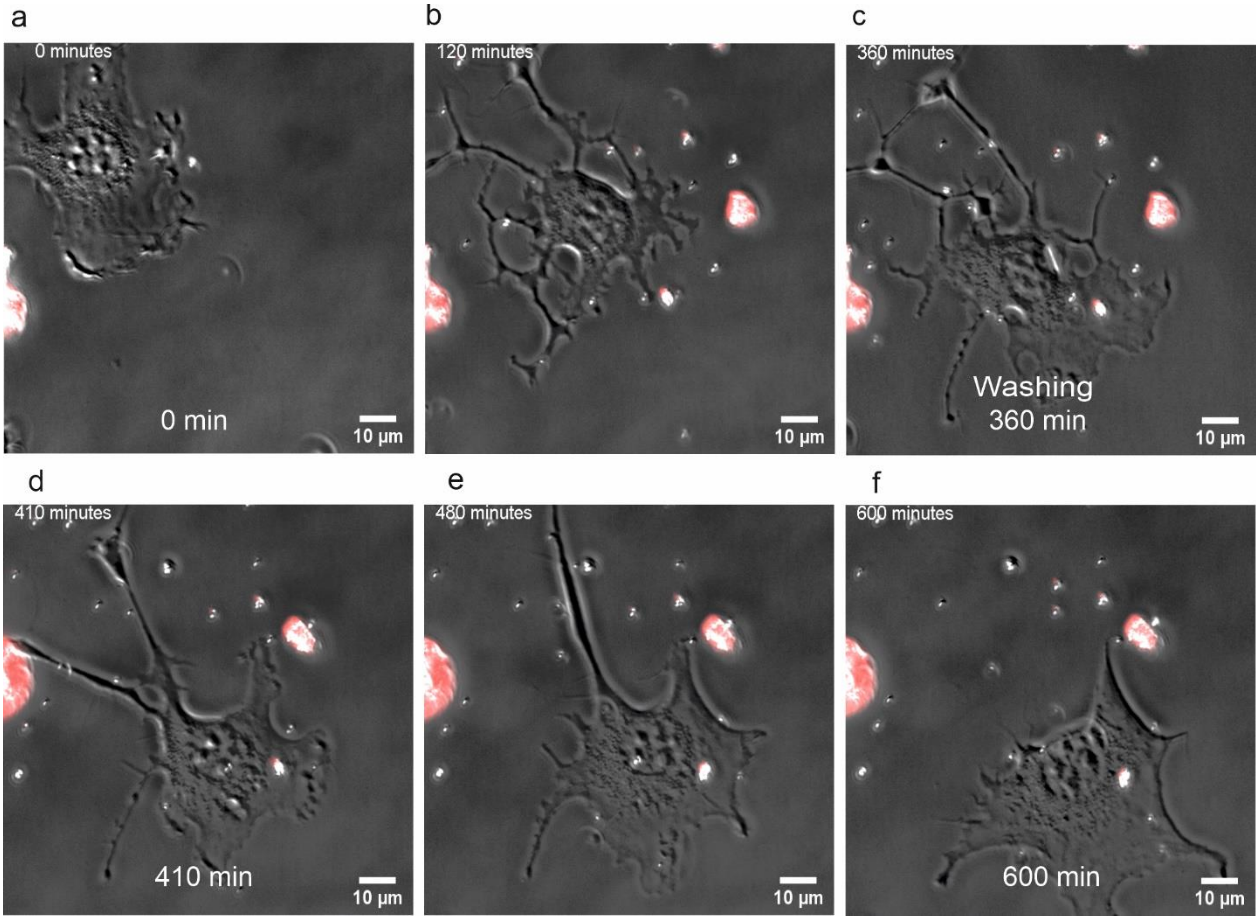

Supplementary Fig. 8. Cells recover after washing out Bleb from the sample. (a-f) show a time-lapse of a cell sample with myosin-II inhibition for 6 h. We can see the cell change its morphology and create tails, but after washing out the Blebbistatin, the cell recovered and retracted the tails with MDs inside. Replications and number of cases are 2 and 20, respectively.

Below, Supplementary Fig. 9a shows the fluorescence intensity of the Myosin-X protein around multiple particles (white arrows) inside the cell. b shows the fluorescence intensity of Myosin-X around the single particle (white arrows) inside the cell. By comparing two panels -a and b - we can see that the amount of Myosin-X around the particles increases when the number of particles increased.

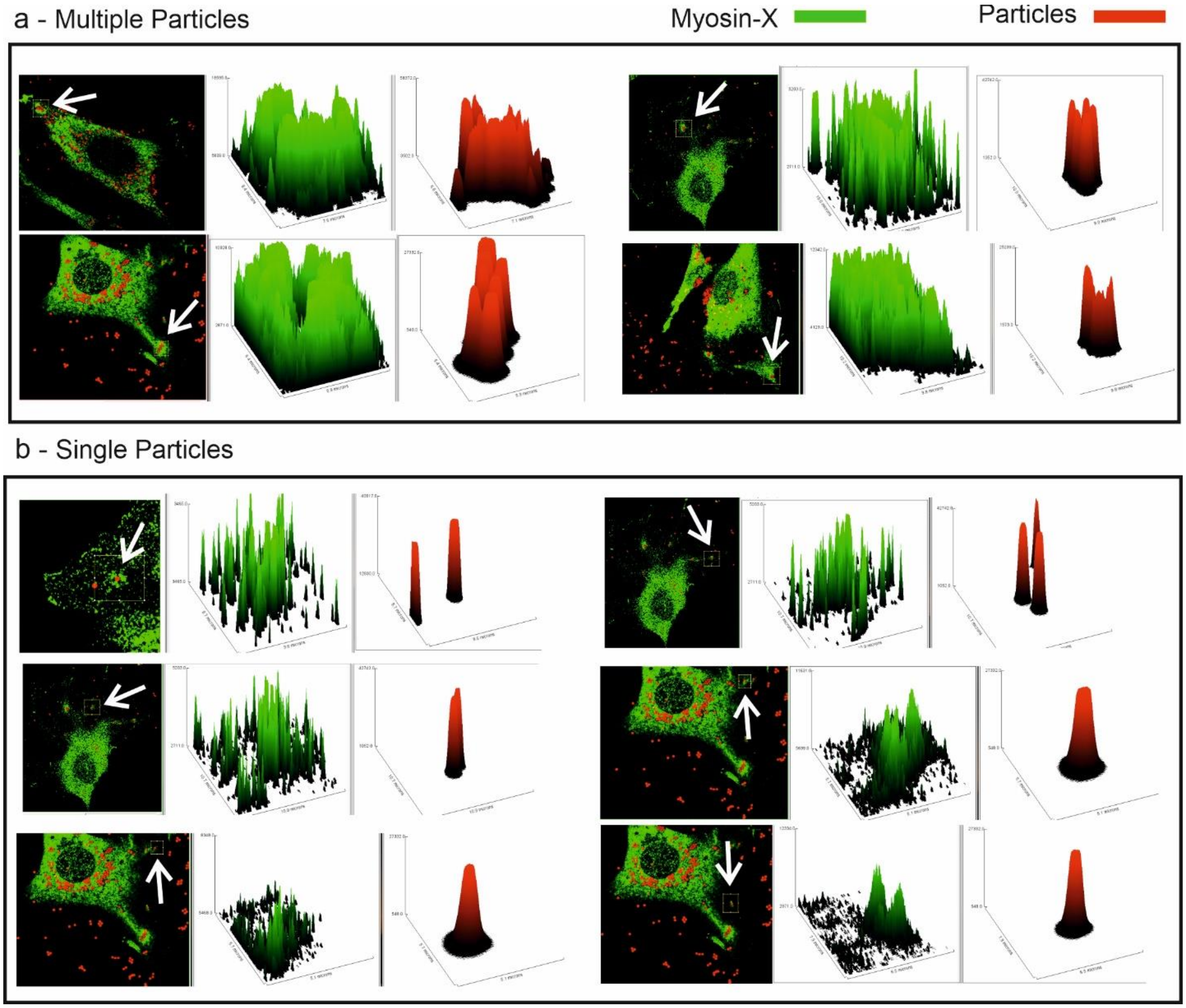

Supplementary Fig. 9. Amount of myosin-X around particles increases with the amount of particles. (a) Fluorescence intensity of myosin-X around multiple particles (white arrows) inside the cell. (b) Fluorescence intensity of myosin-X around the single particle (white arrows) inside the cell. A comparison between panels a and b shows that the amount of myosin-X around the particles increases with the number of particles.

As we can see in a z-stack in Supplementary Fig. 10 when we inhibited myosin-II with Blebbistatin, cells changed their morphology and created long extension and vesicle-like structures. We can see that MDs can

be inside those vesicles (actin in blue, MD in red, myosin-X in green). Also, it is clear that myosin-X also accumulates in those vesicles.

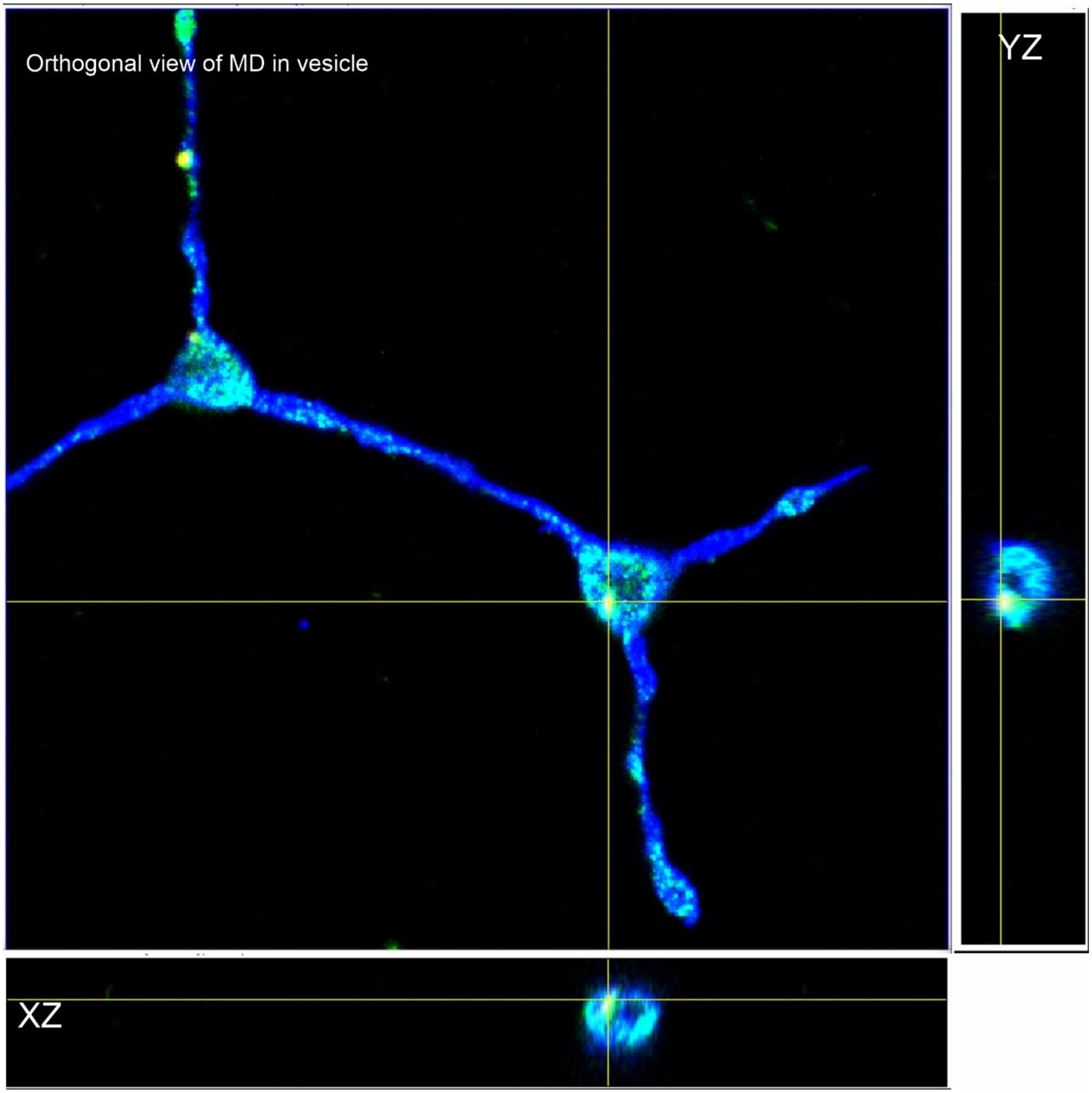

Supplementary Fig. 10. Microdiamonds and myosin-X within a vesicle in a Bleb-tread cell. Orthogonal view of a microdiamond (MD) in one vesicle after myosin-II inhibition shows MD inside the vesicle and also myosin-X is accumulated in that area around the particles within the vesicle. In this figure, actin is in blue, MD is in red, and myosin-X is in green.

Oxygenated MDs exhibit similar behavior as carboxylated MDs

Our experiments showed (Fig. 5a) a greater accumulation of myosin-X in the areas containing carboxylated MDs with microtubules. Another example of the colocalization between myosin-X and microtubules for cells interacting with oxygenated MDs is shown in Supplementary Fig. 11. There were more microtubules in areas 1, 2, and 3 with MDs, and consequently, there were more myosin-X in these areas than in the other areas in the cell. There was no myosin-X in the areas even with more actin filaments. In this study, we selected more spread cells, as it is easier to detect the effects in such cells than in smaller cells with compact cytoskeletons.

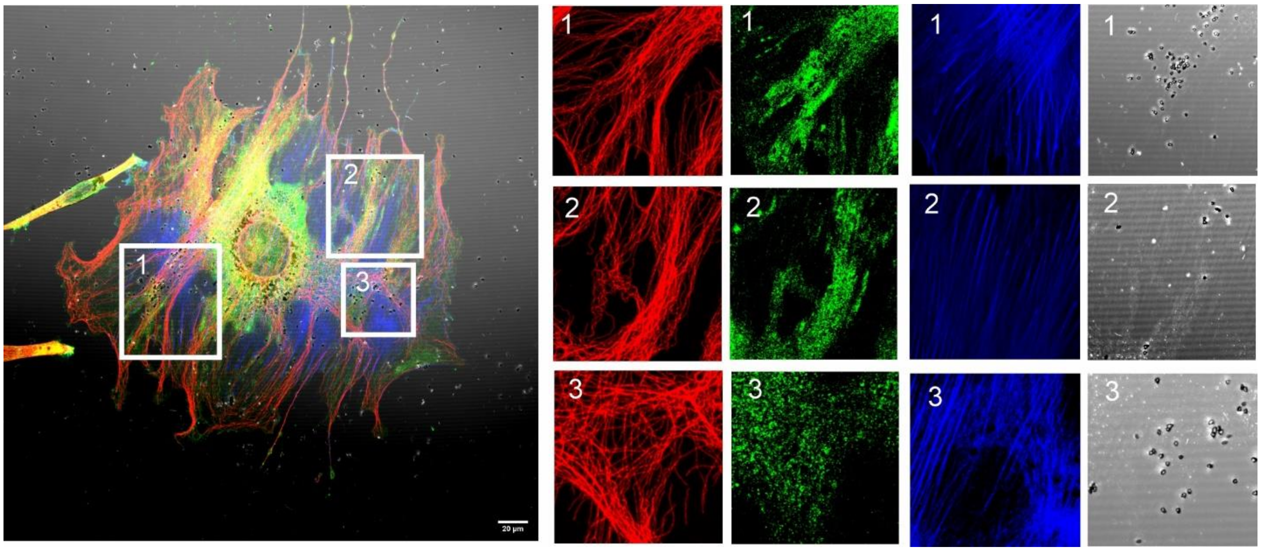

Supplementary Fig. 11. Myosin-X and microtubules show colocalization in the areas where particles are present. Colocalization of myosin-X (green) and microtubules (red) in the presence of oxygenated microdiamonds. One can see that despite the presence of actin filaments (blue) in three selected areas, myosin-X is mostly colocalized with the microtubules,

In Supplementary Fig. 12 we can see separate channels of Myosin-X, microtubules, actin, and MDs for Figure 5 (a). Here, we can see (b) Myosin-X accumulation in the areas with particles and also the track of this protein along the microtubule structure is visible.

a

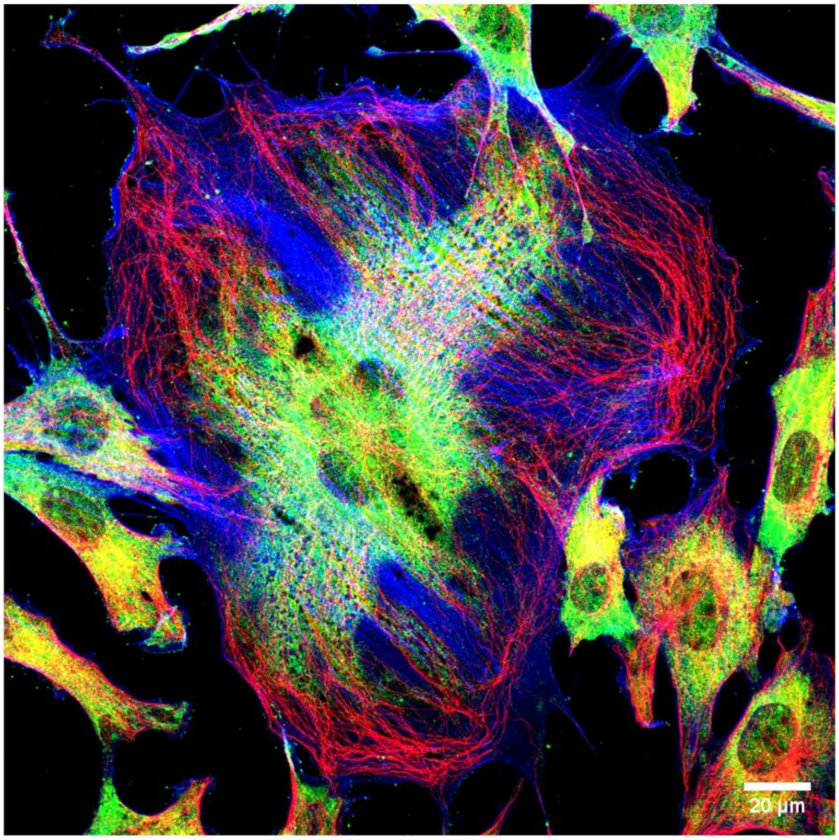

b

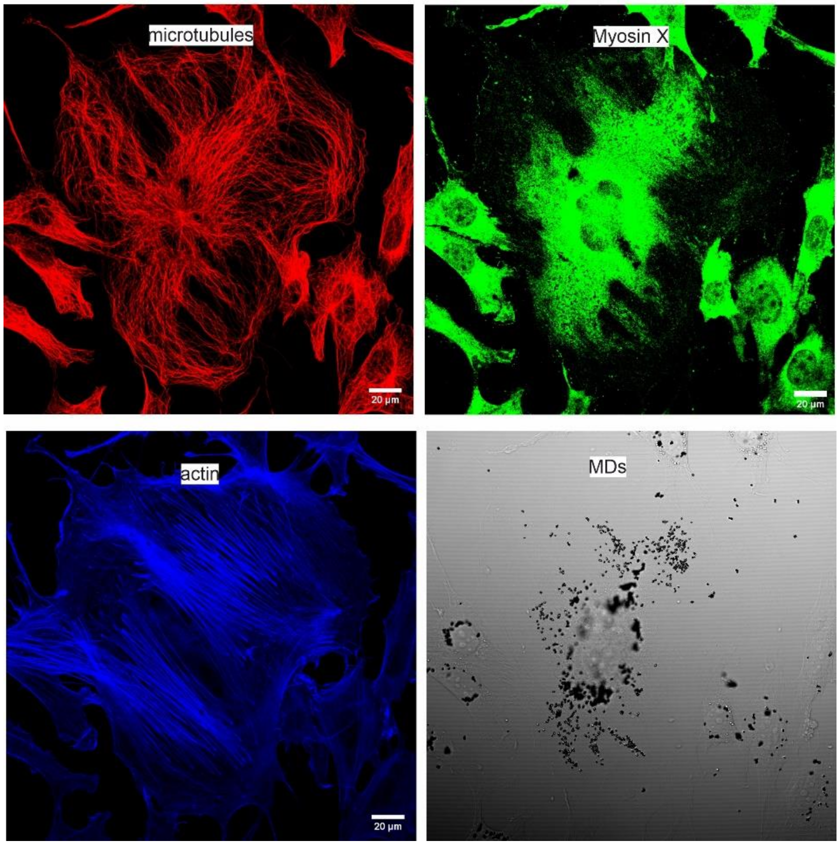

Supplementary Fig. 12. Myosin-X colocalization with microtubules in areas containing particles. Panel (a) shows merged channels of microtubules, myosin-X and actin, and panel (b) shows separate channels of Fig.5. (a) in the main text for microtubules, actin, myosin-X and MDs.

Supplementary Fig. 13 shows separate color channels with myosin-X and microtubules shown in composite Figure 5 (d). Here, we can see myosin-X accumulation in the areas with particles, and also the track of this protein along microtubule structure (i) is visible.

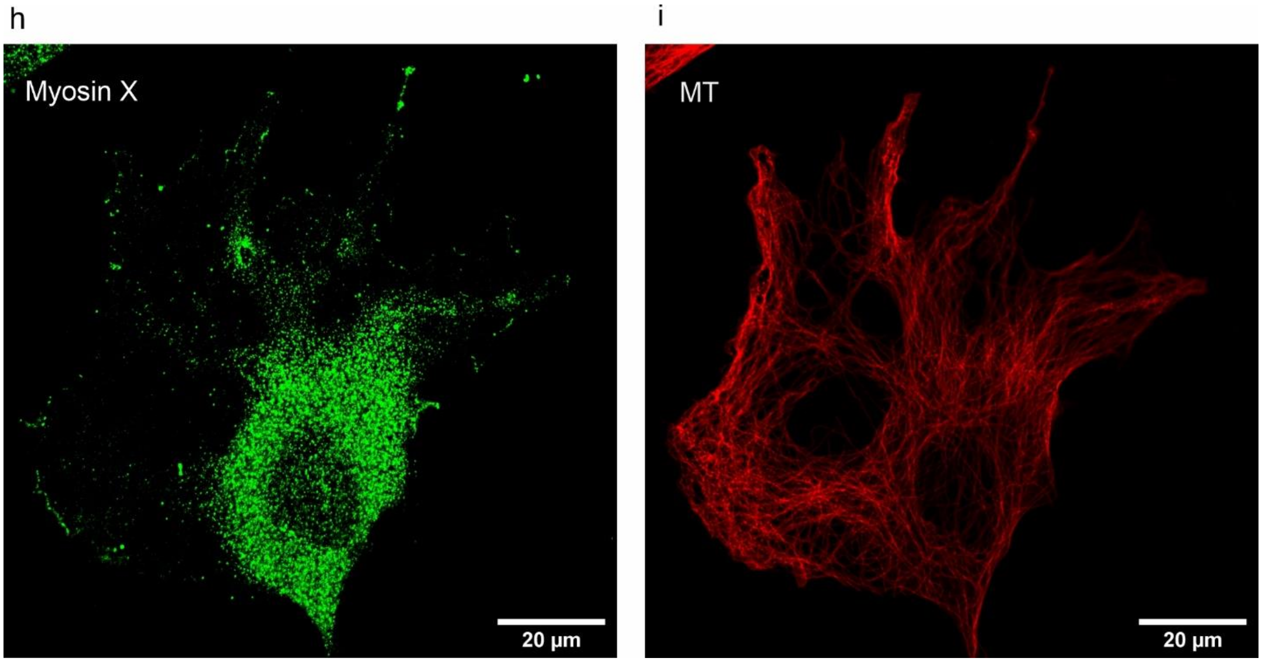

Supplementary Fig. 13. Myosin-X shows traces on microtubules. Separated channels for Fig. 5 d. In (h) and (i) we can see Myosin-X and MT colocalize in the presence of the particles.

Similar sedimentation of MDs and LBs on the surface of the glass bottom dish

As shown in Fig. 2a, cells uptake more MDs, and one factor influencing this process could be the sedimentation rate of both particles. The sedimentation of the particles was observed via live-cell imaging for 14 h. Selected time lapse frames are shown in Supplementary Fig. 14. We observed that within 14 h, both MDs and LBs were weakly attached to the surface. There were slightly more MDs sedimented than LBs, which could be attributed to the higher density of MDs (3.52 g/cm^3) than of LBs (1.05 g/cm^3). In some cases, there were more LBs sedimented on the surface than MDs. The time course of this process is shown in Supplementary Video 11.

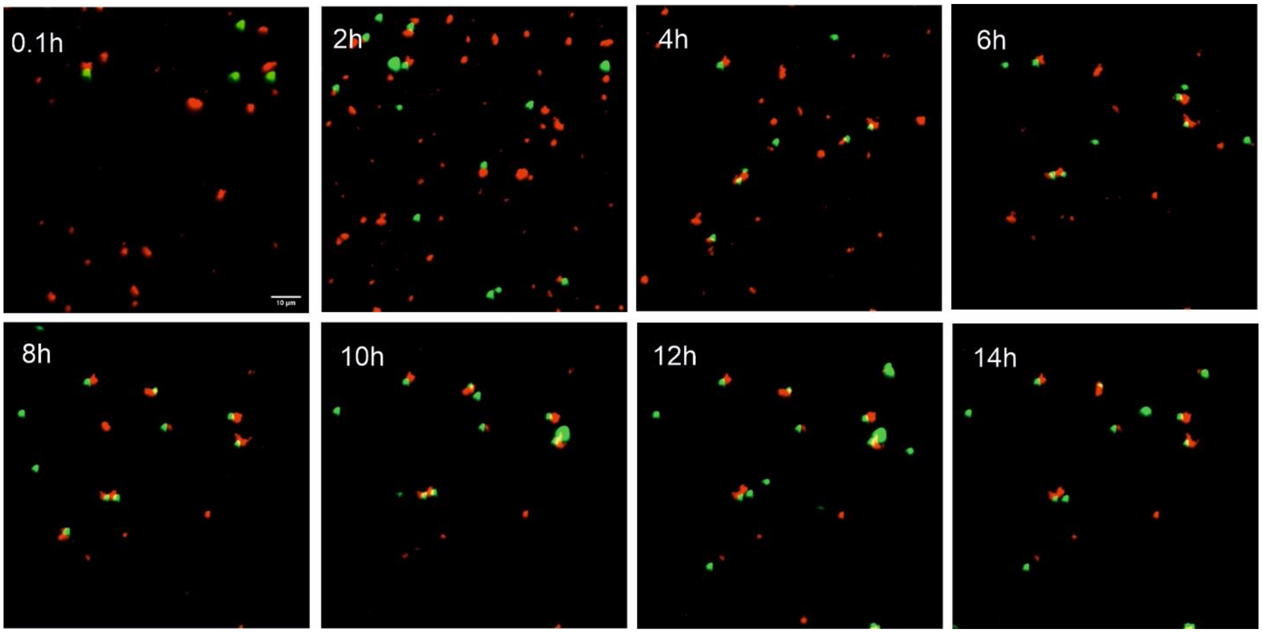

Supplementary Fig. 14. Sedimentation of microdiamonds and latex beads. Investigation of the sedimentation of microdiamonds (MDs) and latex beads (LBs) via live-cell imaging over 14 h. The results show that owing to their higher density, MDs show slightly more sedimentation than LBs but there are no significant differences. Replications and number of cases are 2 and 20, respectively.

MDs do not cause autophagy

As we discussed earlier, our results from the cell samples interacting with MDs at concentrations of up to 100 μg/mL do not show any sign of apoptosis or changes in the morphology of MEF cells. Herein, we investigated the activation of autophagy triggered by MDs and LBs in cells at a concentration of 40 μg/mL. We prepared cell samples with and without particles and stained them for autophagy-related protein LC3B. Between the samples with particles and control, we did not notice any sign of autophagy. Supplementary Fig. 15 shows the autophagy marker after 24 h of incubation of the cells with 40 μg/mL MDs (a), with 40 μg/mL LBs (b), and control sample without particles (c).

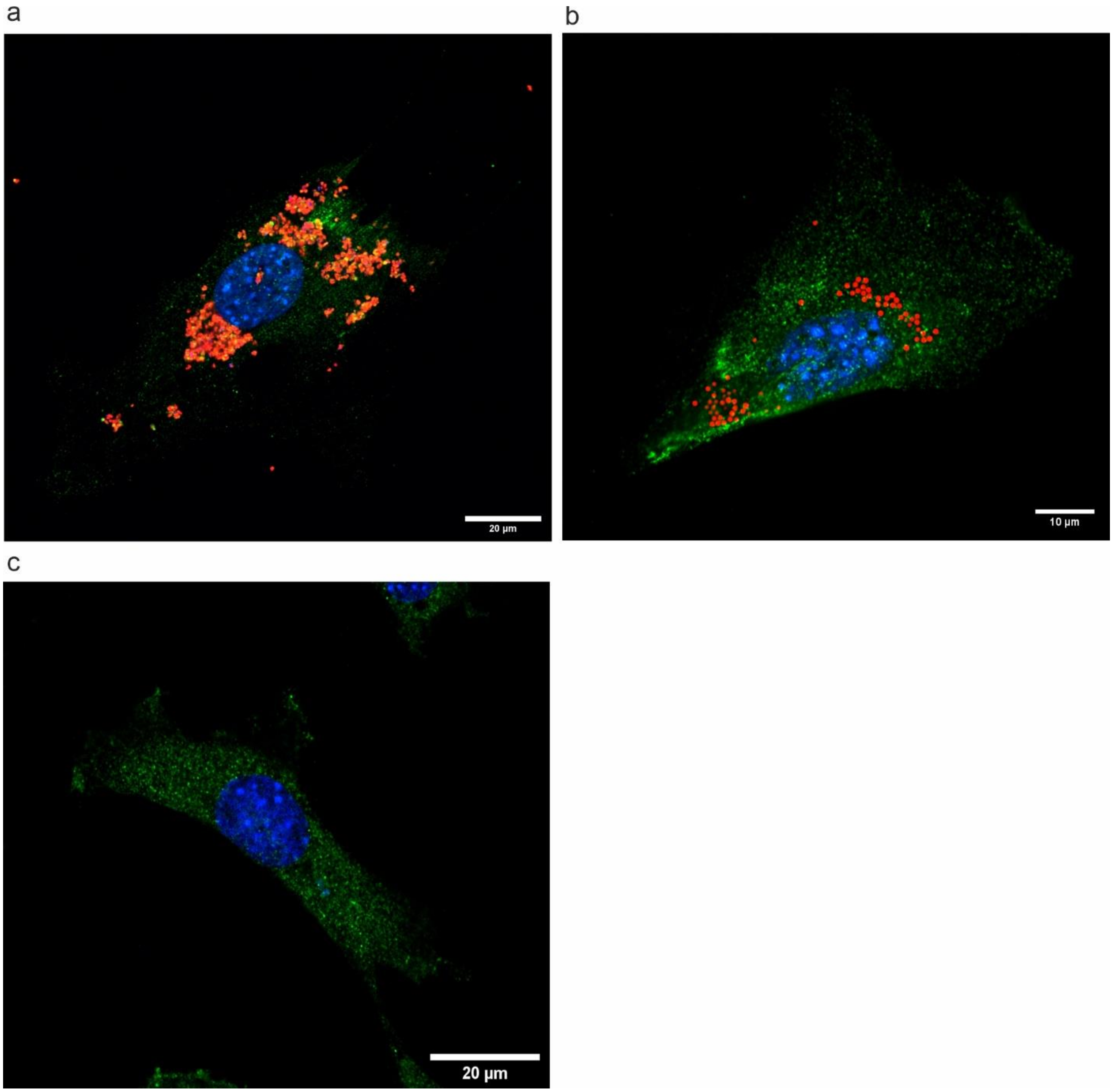

Supplementary Fig. 15. High concentration of particles inside the cell does not cause the autophagy of cells. Autophagy reaction of cells to a high concentration (40 µg/mL) of microdiamonds (a) and latex beads (b). For both particles, no remarkable increase in the LC3B level is detected. (c) Control sample of LC3B without particles. Replications and number of cases are 3 and 30, respectively.

Image Adjustment:

In order to decrease the background signal, for all of the images used in this paper we adjusted the contrast and channel's intensity to clearly present the results. One representative raw image of a cell, fixed and stained for actin (blue), microtubules (red), and myosin-X (green) can be seen in Supplementary Fig. 16, where (a) is the raw image and (b) is the image after adjustment.

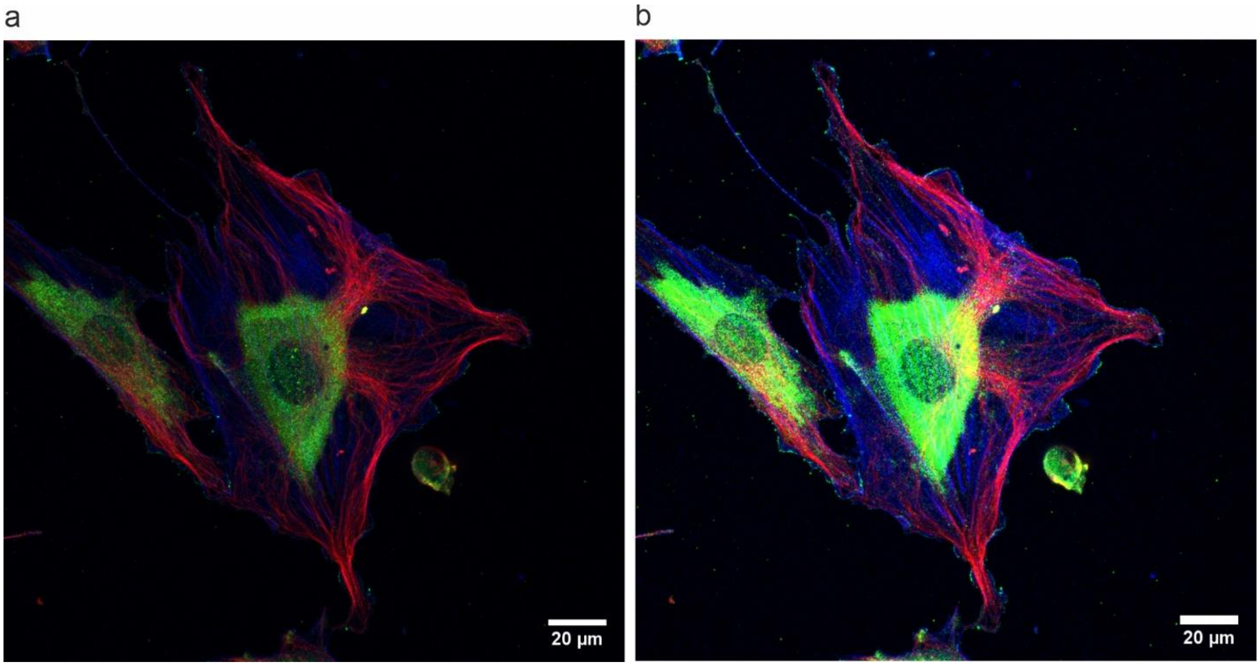

Supplementary Fig. 16. Example of image adjustment. Raw image (a) of a cell stained for actin (blue), microtubules (red), and myosin-X (green) and adjusted same image (b).

Supplementary Note

Moving vesicles

Our time lapse experiments showed (Fig. 3b) that not all vesicles contained particles and entered the cells. This might be attributed to the cell movement and the combination of this movement with the complex morphology caused by the Bleb treatment. In some cases, the vesicles containing MDs moved along the tails but could neither reach the main body nor enter the cells. Our results do not indicate whether the transport of particles with vesicles is a cell-controlled process or a random activity; regardless of such, it can affect cell–particle interaction and increase the number of particles inside cells.

Characterization of particles

Characterization of microdiamonds (MDs)

Provided by the manufacturer, the 1 µm particles (NDNV1umCOOH, Adamas Nanotechnologies, Raleigh, NC USA) were negatively charged with a nominal zeta potential of -30 mV and a Z-average size of ca. 730 nm based on electrophoretic light scattering (ELS) and dynamic light scattering (DLS) measurements (Zetasizer Advance, Malvern Panalytical, UK), respectively.

Referring to the manufacturer description, particles will be unstable in buffer media with high ionic strengths (e.g., 0.5X or 1X PBS), and due to particle size, they do not show long-term stability in any typical media and particles will sediment after several hours or a day.

Moreover, in terms of surface termination, **oxygenated** MDs can exhibit various configurations, including but not limited to carbonyl (C=O), ether (C-O-C), hydroxyl (C-O-H), and even diester (C-O-O-C) arrangements. Regarding **carboxyl** group functionalization, the surface of MDs can undergo modifications resulting in carboxyl groups (COOH). These modifications involve the introduction of carboxyl functional entities onto the diamond surface, which can influence the chemical interactions and reactivity of the material. Due to changes in surface chemistry and bioconjugation capabilities, the specific functional groups present in carboxylated and oxygenated MDs can influence their behavior and interactions in biological samples. Furthermore, carboxyl functional groups exhibit different features compared to oxygen terminations, as they possess the capability to engage in peptide bond formation with amino parts. Additionally, carboxyl groups typically occupy a minor fraction of the diamond surface; nonetheless, they are commonly used due to their facile reactivity for bioconjugation purposes.

Characterization of Red Latex Beads (LBs)

Surface modification: Carboxylate-modified red Microspheres
Material: Polystyrene
Label (Ex/Em): Red fluorescent (580/605 nm)
Nominal bead diameter: 1.0 µm
Composition: Solids, 2%
Form: aqueous suspension

Characterization of Green Latex Beads (LBs)

Surface modification: Carboxylate-modified green Microspheres
Material: Polystyrene
Label (Ex/Em): yellow-green fluorescent (470/505 nm)

Mean Diameter: 0.90 - 1.10 micron
Composition: Solids, 2.5%
Form: aqueous suspension

Utilization of Different Particles

It is worth to mention that the utilization of carboxylated (COOH) and oxygenated MDs, with and without NVs, and carboxylated latex beads in our study served different purposes.

We chose to utilize COOH MDs in our study for two reasons, first due to their reactivity for bioconjugation purposes they are commonly used. Secondly, our previous expertise with them allowed us to employ those MDs to study the cell-MDs interaction. Oxygenated MDs were employed to compare the influence of different MDs surface functional groups on the observed phenomena of MDs uptake and myosin-X results. Also, to have consistency and proper control experiments, in every case in which we used COOH MDs, we also used LBs with COOH functionalization. It is worth mentioning that our main work in this paper was done with COOH MDs and we only used oxygenated MDs for comparison, to check whether the observations of myosin-X and uptake are only happening for COOH MDs, or we can see the same behavior for oxygenated MDs.

In the context of utilizing non-fluorescent MDs (without NV centers), our choice was influenced by the limited number of available color channels on the confocal microscope. In certain cell immunostaining experiments, distinct fluorescent antibodies were employed to identify various cellular components such as actin (depicted in blue), microtubules (represented in red), endosomes, or myosin-X (displayed in green). As a result, the confocal microscopy setup needed to allocate the green, blue, and red channels for these specific components, which was essential for the intended investigations. To ensure the detection of MDs without causing interference with the emission spectra of other fluorescent markers, we opted to utilize MDs without color centers. These MDs were detected using gray channel, as illustrated in Figures 5 and 6. This strategic approach enabled us to achieve simultaneous cell cross-staining while successfully detecting MDs within the cellular context.
