# Supplementary material for: Cell-particles interaction – selective uptake and transport of microdiamonds
Source: Commun Biol. 2024 Mar 13;7:318. doi: 10.1038/s42003-024-05974-4 (PMC10937934; doi:10.1038/s42003-024-05974-4)
Supplement: Supplementary file 2 — Description of Additional Supplementary Files [file 42003_2024_5974_MOESM2_ESM.pdf]

Description of Additional Supplementary Files

Video Files Legends

File name: Supplementary Video 1

Description: Time-lapse of EGFP-F-tractin-transfected cell, sensing microdiamonds sedimented on the dish surface with filopodia and uptaking them over time.

File name: Supplementary Video 2

Description: Live imaging of a cell interaction with latex beads and microdiamonds. The cell uptakes only diamond particles at the end.

File name: Supplementary Video 3

Description: An example of an active transport of microdiamonds along actin filaments, visualized by live imaging of EGFP-F-tractin-transfected cell.

File name: Supplementary Video 4

Description: Zoomed part of Video 2d showing an active transport of microdiamond along the actin filament.

File name: Supplementary Video 5

Description: Live imaging of cells treated with Blebbistatin. After inhibition of myosin-II activity, cells still continue the search and uptake of diamonds particles.

File name: Supplementary Video 6

Description: Live cell imaging of cell changing its morphology by creating long extensions with vesicles, after Blebbistatin treatment. Some of the newly formed vesicles, containing particles inside them, can move along the tails to the cell body.

File name: Supplementary Video 7

Description: Live imaging shows the interaction between cells and latex beads.

File name: Supplementary Video 8

Description: Interaction between a cell and latex beads after Blebbistatin treatment is similar to untreated cells.

File name Supplementary Video 9

Description: Inhibition of myosin-II activity did not significantly affect the interaction between cells and latex beads.

File name: Supplementary Video 10

Description: An example of the selective uptake of microdiamonds over latex beads. One can notice that although the cell is in touch with several latex beads, in the end, cell uptakes only the diamond particles.

File name: Supplementary Video 11

Description: Live imaging of microdiamonds and latex beads sedimentation. One can see slightly more sedimented microdiamonds than latex beads.

File name: Supplementary Video 12

Description: Live imaging of a cell sample treated with Blebb and after 6 h, by washing the Bleb out of the sample, cells can recover their morphology and continue probing.
