# Supplementary material for: Cell-particles interaction – selective uptake and transport of microdiamonds
Source: Commun Biol. 2024 Mar 13;7:318. doi: 10.1038/s42003-024-05974-4 (PMC10937934; doi:10.1038/s42003-024-05974-4)
Supplement: Supplementary file 15 — Reporting Summary [file 42003_2024_5974_MOESM15_ESM.pdf]

nature portfolio

Corresponding author(s): Zenon Rajfur

Last updated by author(s): Feb 13, 2024

Reporting Summary

Nature Portfolio wishes to improve the reproducibility of the work that we publish. This form provides structure for consistency and transparency in reporting. For further information on Nature Portfolio policies, see our Editorial Policies and the Editorial Policy Checklist.

Statistics

For all statistical analyses, confirm that the following items are present in the figure legend, table legend, main text, or Methods section.

n/a	Confirmed	
☐	☒	The exact sample size (n) for each experimental group/condition, given as a discrete number and unit of measurement
☐	☒	A statement on whether measurements were taken from distinct samples or whether the same sample was measured repeatedly
☒	☐	The statistical test(s) used AND whether they are one- or two-sided<br>*Only common tests should be described solely by name; describe more complex techniques in the Methods section.*
☒	☐	A description of all covariates tested
☒	☐	A description of any assumptions or corrections, such as tests of normality and adjustment for multiple comparisons
☒	☐	A full description of the statistical parameters including central tendency (e.g. means) or other basic estimates (e.g. regression coefficient) AND variation (e.g. standard deviation) or associated estimates of uncertainty (e.g. confidence intervals)
☒	☐	For null hypothesis testing, the test statistic (e.g. F, t, r) with confidence intervals, effect sizes, degrees of freedom and P value noted<br>*Give P values as exact values whenever suitable.*
☒	☐	For Bayesian analysis, information on the choice of priors and Markov chain Monte Carlo settings
☒	☐	For hierarchical and complex designs, identification of the appropriate level for tests and full reporting of outcomes
☒	☐	Estimates of effect sizes (e.g. Cohen's d, Pearson's r), indicating how they were calculated

Our web collection on statistics for biologists contains articles on many of the points above.

Software and code

Policy information about availability of computer code

Data collection	ZEN 2.6 (blue edition) by Zeiss, ZEN 2012 8.1.0.484 (black edition) by Zeiss
Data analysis	ZEN 3.4 (blue edition) by Zeiss<br>ImageJ-win64

For manuscripts utilizing custom algorithms or software that are central to the research but not yet described in published literature, software must be made available to editors and reviewers. We strongly encourage code deposition in a community repository (e.g. GitHub). See the Nature Portfolio guidelines for submitting code & software for further information.

Data

Policy information about availability of data

All manuscripts must include a data availability statement. This statement should provide the following information, where applicable:

- Accession codes, unique identifiers, or web links for publicly available datasets
- A description of any restrictions on data availability
- For clinical datasets or third party data, please ensure that the statement adheres to our policy

All data reported in this paper are available from the corresponding authors upon request.

Research involving human participants, their data, or biological material

Policy information about studies with human participants or human data. See also policy information about sex, gender (identity/presentation), and sexual orientation and race, ethnicity and racism.

Reporting on sex and gender	N/A
Reporting on race, ethnicity, or other socially relevant groupings	N/A
Population characteristics	N/A
Recruitment	N/A
Ethics oversight	N/A

Note that full information on the approval of the study protocol must also be provided in the manuscript.

Field-specific reporting

Please select the one below that is the best fit for your research. If you are not sure, read the appropriate sections before making your selection.

☒ Life sciences ☐ Behavioural & social sciences ☐ Ecological, evolutionary & environmental sciences

For a reference copy of the document with all sections, see nature.com/documents/nr-reporting-summary-flat.pdf

Life sciences study design

All studies must disclose on these points even when the disclosure is negative.

Sample size	No sample size calculation was performed.
Data exclusions	Fig.1 (A-E) - images of cells not containing microdiamonds excluded from analysis; Fig.5 - images of cells not well spread were excluded; Fig. 6 B - images of cells without EEA1 staining were excluded. All other sets of images were analyzed.
Replication	Most of the stained cells images were made in at least triplicate or more with at least 15 different fields of view. Live cell images shown on Fig. 2 B,C and Supplementary Fig.6 are from four replicate and 14 cells because of the difficulty of finding the cell with the latex beads and microdiamonds close to each other and close to the cell. Fig. 3 A,B shows the results from one sample and multiple fields of view (in total triplicate and 20 cases).<br>Information of replication and number of cases for each panel is noted in the manuscript in each Figure legend.<br><br>Number of replications (R) and cases (C) for each Fig (all panels) are as follows:<br>(Fig 1) R: 9 C: 60<br>(Fig 2) R: 11 C: 90<br>((Fig 3) R: 9 C: 120<br>(Fig 4) R: 13 C: 90<br>(Fig 5) R: 7 C: 33<br>(Fig 6) R: 4 C: 33
Randomization	Not applicable.
Blinding	The blinding was not relevant to our study because we did not have any preconceived expectations of the experimental results at any stage of the study. Some of our experimental results, for example cellular actin, microtubules architecture or EEA1 and Myosin X localisation in normal cells (without particles) are known and the experiments involving such measurements were performed in order to check the quality of our procedures. Since there are novel findings in our work, the personal bias of the observer had no influence on data selection.

Reporting for specific materials, systems and methods

We require information from authors about some types of materials, experimental systems and methods used in many studies. Here, indicate whether each material, system or method listed is relevant to your study. If you are not sure if a list item applies to your research, read the appropriate section before selecting a response.

Materials & experimental systems

n/a	Involved in the study	
☐	☒	Antibodies
☐	☒	Eukaryotic cell lines
☒	☐	Palaeontology and archaeology
☒	☐	Animals and other organisms
☒	☐	Clinical data
☒	☐	Dual use research of concern
☒	☐	Plants

Methods

n/a	Involved in the study	
☒	☐	ChIP-seq
☒	☐	Flow cytometry
☒	☐	MRI-based neuroimaging

Antibodies

Antibodies used

CN: Catalog Number
Monoclonal Anti-α-Tubulin antibody (Sigma, CN:T9026, clone DM1A, ascites fluid)
Anti-Myosin IIA (Sigma, CN:M8064, clone MY-32, ascites fluid)
Anti-MYO10 antibody (Sigma, CN:HPA024223)
Anti-EEA1 antibody (Cell signaling, CN:C45B10)
Anti-EEA1 antibody (Sigma, CN:E7659, clone EEA1-N19)
LC3B Polyclonal Antibody (Invitrogen, PA5-32254)

Secondary antibodies from Invitrogen:
Goat anti-Rabbit IgG (H+L) Cross-Adsorbed Secondary Antibody, Alexa Fluor™ 647 (CN:A21244)
Goat anti-Mouse IgG (H+L) Highly Cross-Adsorbed Secondary Antibody, Alexa Fluor™ Plus 647 (CN:A32728)
Goat anti-Rabbit IgG (H+L) Cross-Adsorbed Secondary Antibody, Pacific Blue™ (CN:P10994)
Goat anti-Rabbit IgG (H+L) Highly Cross-Adsorbed Secondary Antibody, Alexa Fluor™ Plus 488 (CN: A32731)
Goat anti-Mouse IgG (H+L) Highly Cross-Adsorbed Secondary Antibody, Alexa Fluor™ Plus 488 (CN: A32723)

Validation

Sigma : All Prestige Antibodies Powered by Atlas Antibodies are developed and validated by the Human Protein Atlas (HPA) project and as a result, are supported by the most extensive characterization in the industry.

Invitrogen : This Antibody was verified by Cell treatment to ensure that the antibody binds to the antigen stated.

Cell Signaling : we adhere to the Hallmarks of Antibody Validation™.

Eukaryotic cell lines

Policy information about cell lines and Sex and Gender in Research

Cell line source(s)	Mouse Embryo Fibroblasts MEF 3T3, ATCC CRL-1658
Authentication	None of the cell lines were authenticated.
Mycoplasma contamination	Mycoplasma testing was done with Hoechst 3322 dye showing no punctate staining around the cells.
Commonly misidentified lines (See ICLAC register)	N/A
